# Beyond DNA Demethylation: Curcumin as a Context-Dependent Remodeler of the Epigenetic Landscape

**DOI:** 10.3390/ijms27219672

**Published:** 2026-10-28

**Authors:** Salvatore Costa, Maria Antonietta Ragusa, Aldo Nicosia

**Affiliations:** 1 Dipartimento di Scienze e Tecnologie Biologiche Chimiche e Farmaceutiche (STEBICEF), Sezione di Biologia Cellulare, Università di Palermo, Viale delle Scienze, Edificio 16, 90128 Palermo, Italy; salvatore.costa@unipa.it (S.C.); maria.ragusa@unipa.it (M.A.R.); 2 Institute for Biomedical Research and Innovation-National Research Council (IRIB-CNR), 90146 Palermo, Italy

**Keywords:** curcumin, DNA methylation, 5-hydroxymethylcytosine, TET enzymes, context-dependent regulation, multi-omics epigenomics, histone modifications, non-coding RNAs, bioavailability, cancer

## Abstract

Curcumin has attracted considerable attention for its pleiotropic effects on cellular signaling, inflammation, metabolism, and cancer biology. A growing body of evidence indicates that these effects coincide with alterations in the epigenetic landscape; however, a causal contribution of such alterations has seldom been established experimentally. Although curcumin has frequently been described as an epigenetic demethylating agent, accumulating evidence suggests more complex, context-dependent mechanisms, which involve DNA methylation, hydroxymethylation, histone modifications, chromatin remodeling, and non-coding RNAs. These changes may affect the accessibility and transcriptional activity of genes involved in cell identity, proliferation, differentiation, inflammation, and therapeutic response. The few genome-wide methylomic and transcriptomic studies available, mostly performed in a single cell line and at micromolar concentrations that exceed those reached in vivo, suggest that curcumin-induced epigenetic changes depend on cellular context, dose, and exposure conditions. This narrative review summarizes evidence published mainly from 2004 onward on the effects of curcumin on DNA methylation and hydroxymethylation, while integrating its interactions with histone modifications, chromatin organization, and non-coding RNA networks. We further discuss how these interconnected mechanisms contribute to the rewiring of gene regulatory programs and may underlie the biological and therapeutic effects of curcumin. Finally, we highlight the current limitations, including the poor bioavailability of curcumin, the non-specific chemistry of the molecule, and the scarcity of integrative multi-omics data, and we discuss what would be required to establish whether curcumin-induced epigenetic changes are relevant at pharmacologically attainable concentrations.

## 1. Introduction

Curcumin is a natural diarylheptanoid and represents the principal curcuminoid recovered from the rhizome of *Curcuma longa* L. Over the years, it has attracted sustained scientific interest because of its reported antioxidant, anti-inflammatory, immunomodulatory and anticancer activities [1,2]. At the molecular level, curcumin has been shown to act on different pathways, including NF-κB, PI3K/AKT, MAPK and Wnt/β-catenin signaling, thus influencing the related gene regulatory network, while several lines of evidence indicate changes in epigenetic regulation, including DNA methylation, histone-modifying enzymes and non-coding RNA expression [2,3,4].

It is known that epigenetic regulation encompasses molecular processes, which include DNA methylation and hydroxymethylation, post-translational histone modifications, nucleosome positioning, chromatin remodeling and regulatory non-coding RNAs [5,6]. Although these mechanisms are often investigated separately, they operate as interconnected regulatory layers influencing the epigenetic landscape so as to define chromatin accessibility, transcriptional and cellular plasticity [5].

The term “landscape” derives from Waddington, who used it to define the progressive restriction of developmental potential during cell fate determination [7]. Nowadays, the concept has acquired a broader meaning and is used to describe the dynamic regulatory architecture through which genetic background, chromatin state, transcriptional networks and environmental signals interact to stabilize or modify cellular identity [5,8,9]. This is especially relevant to pharmacological interventions because modification in the activity/amount of a single epigenetic enzyme may in turn affect multiple regulatory layers so as to produce effects that differ according to genomic and cellular context.

Epigenetic dysregulation stands as a cornerstone of cancer onset and progression. Tumor cells frequently exhibit general losses in DNA methylation alongside localized gains at specific promoters and other regulatory regions. These disruptions intersect with broad alterations in DNA hydroxymethylation, histone marks, enhancer actions, chromatin accessibility, and non-coding RNA networks [5,7,10]. Far from behaving as isolated anomalies, these changes interact dynamically to establish transcriptional profiles that drive tumor survival, proliferation, cellular plasticity, invasion, and evasion of therapeutic agents [10,11]. Consequently, evaluating cancer epigenomes simply through the lens of individual chromatin-modifying enzymes being turned on or off remains an inadequate approach.

The potential to reverse these epigenetic changes has fueled a major push towards epigenetic drugs. In clinical settings, established epidrugs include DNA methyltransferase and histone deacetylase inhibitors, which have shown particular utility against specific hematological malignancies [12]. Even so, how these agents function illustrates the complexity of pharmacological epigenetic control. Blocking a single enzymatic class often triggers widespread, context-dependent effects that reshape transcription, differentiation, immune signaling, and survival pathways. Such complexity has driven interest toward natural compounds capable of hitting multiple pieces of the epigenetic machinery at once, though validating their selectivity, effective dosing, and actual clinical utility still demands rigorous analysis [3,4,12].

Among dietary options investigated for their epigenetic properties, curcumin has received some of the closest attention. Initial research focused heavily on its impact on single targets, namely DNMT1, specific histone acetyltransferases and deacetylases, or individual oncogenic and tumor-suppressive microRNAs [3,4]. These early findings led many to categorize curcumin routinely as a natural DNMT or HDAC inhibitor. However, this viewpoint might be overly simplistic. Shifts in the level or function of an epigenetic enzyme do not automatically translate to a uniform modification of its target mark across the entire genome. Instead, the actual biological outcome hinges on the precise genomic regions involved, the existing chromatin baseline, available transcription factors, and the specific identity or state of the cell being targeted [13].

Data from recent genome-scale investigations point toward a far more intricate scenario. For instance, whole-genome bisulfite sequencing of MDA-MB-231 triple-negative breast cancer cells treated with the curcumin derivatives, namely ST08 and ST09, revealed a diverse mix of hypermethylated and hypomethylated sites, spanning promoter CpG islands as well as gene bodies [14] (synthetic derivatives, not native curcumin). Integrating these findings with RNA sequencing highlighted clear, derivative-specific links between methylation shifts, gene expression, and downstream biological pathways in which ST08 altered processes tied mainly to the extracellular matrix, while ST09 primarily impacted on cell-cycle pathways [14]. Given that these compounds are synthetic derivatives, not native curcumin, with cytotoxic activity in the nanomolar range, these outcomes cannot be mapped directly onto native curcumin. This study nonetheless indicates that exposure to these derivatives can trigger a selective, bidirectional remodeling of the DNA-methylation landscape, rather than causing a blanket, indiscriminate clearing of genome-wide methylation.

A complementary perspective emerged from the analysis of doxorubicin-resistant MCF-7 cells treated with native curcumin. In this model, reduced-representation bisulfite sequencing, RT-qPCR of selected genes and protein assays indicated that curcumin treatment was associated with differential methylation of selected genomic regions and with changes in transcriptional and cellular programs [15]. The results showed a predominantly demethylating effect concentrated at defined genomic regions rather than a uniform genome-wide change, in association with changes in phenotypes linked to acquired drug resistance, and a partial reversal of resistance-associated methylation [15]. These findings are particularly relevant because therapy-resistant cells are maintained by interconnected genetic, transcriptional and epigenetic mechanisms rather than by a single reversible lesion.

Current evidence, therefore, suggests that curcumin should not be described exclusively as an inhibitor of individual epigenetic enzymes or as a general DNA-demethylating compound. A working hypothesis is that curcumin influences the epigenetic landscape in a context-dependent manner, with effects determined by compound formulation, concentration, exposure time, cell lineage, basal epigenetic state and disease phenotype [16,17]. Under this model, modulation of DNMTs, histone-associated enzymes and non-coding RNAs represents only one component of a broader regulatory response involving locus-specific epigenetic changes and transcriptional-network reorganization.

This distinction matters because much of the available literature remains based on candidate genes, selected microRNAs, measurements of global methylation or expression analysis of individual chromatin regulators [3,4]. Direct evidence derived from WGBS, RRBS, hydroxymethylome profiling, chromatin-accessibility assays or integrated epigenomic analyses is still comparatively limited. Also, curcumin derivatives, nanoformulations and native curcumin should not be considered interchangeable, as differences in chemical stability, cellular uptake, metabolism and potency may result in distinct molecular and epigenetic outcomes.

In this review, we critically examine evidence published from 2004 onward concerning the effects of curcumin on the epigenetic landscape, in order to include landmark studies when necessary to establish the mechanistic background. Particular attention is devoted to DNA methylation and hydroxymethylation, histone-associated regulation, non-coding RNA networks and the limited but emerging genome-wide and multi-omics evidence. Rather than presenting curcumin as a universally acting natural epidrug, we discuss the possibility that its effects are better interpreted through a working model of context-dependent epigenetic network rewiring, which remains a hypothesis rather than an established mechanism, in which multiple regulatory layers are perturbed and reorganized according to the biological state of the exposed cell. Relevant studies were identified through databases search using combinations of terms, such as “curcumin”, “curcuminoid”, “epigenetic”, “DNA methylation”, “hydroxymethylation”, “histone”, “microRNA”, “WGBS” and “RRBS. Reports were selected by the authors on the basis of relevance to the topic; no formal systematic or quantitative synthesis was performed, and the statements on the scarcity of data should be read with this limitation in mind. Throughout the review, “inhibition” denotes reduced enzymatic activity measured in vitro (cell-free assays), whereas reduced mRNA or protein levels of an enzyme in cells are described as “downregulation”; the two are mechanistically distinct and are not used interchangeably. One of the genome-wide studies discussed (Poma et al., Section 3.5) was co-authored by all the authors of this review; it is described and appraised with the same criteria applied to the other reports.

## 2. From Epigenetic Enzymes to Epigenetic Landscapes

For a long time, epigenetic regulation was studied one enzyme class at a time. DNMTs, HATs, HDACs, HMTs, histone demethylases were each approached more or less in isolation, on the assumption that modulating one target pharmacologically would produce a reasonably predictable effect. It was a reductionist view but not a wasted one: it gave cancer epigenetics its footing and produced the first clinically approved epidrugs, DNMT and HDAC inhibitors among them [18,19].

**Figure 1:**
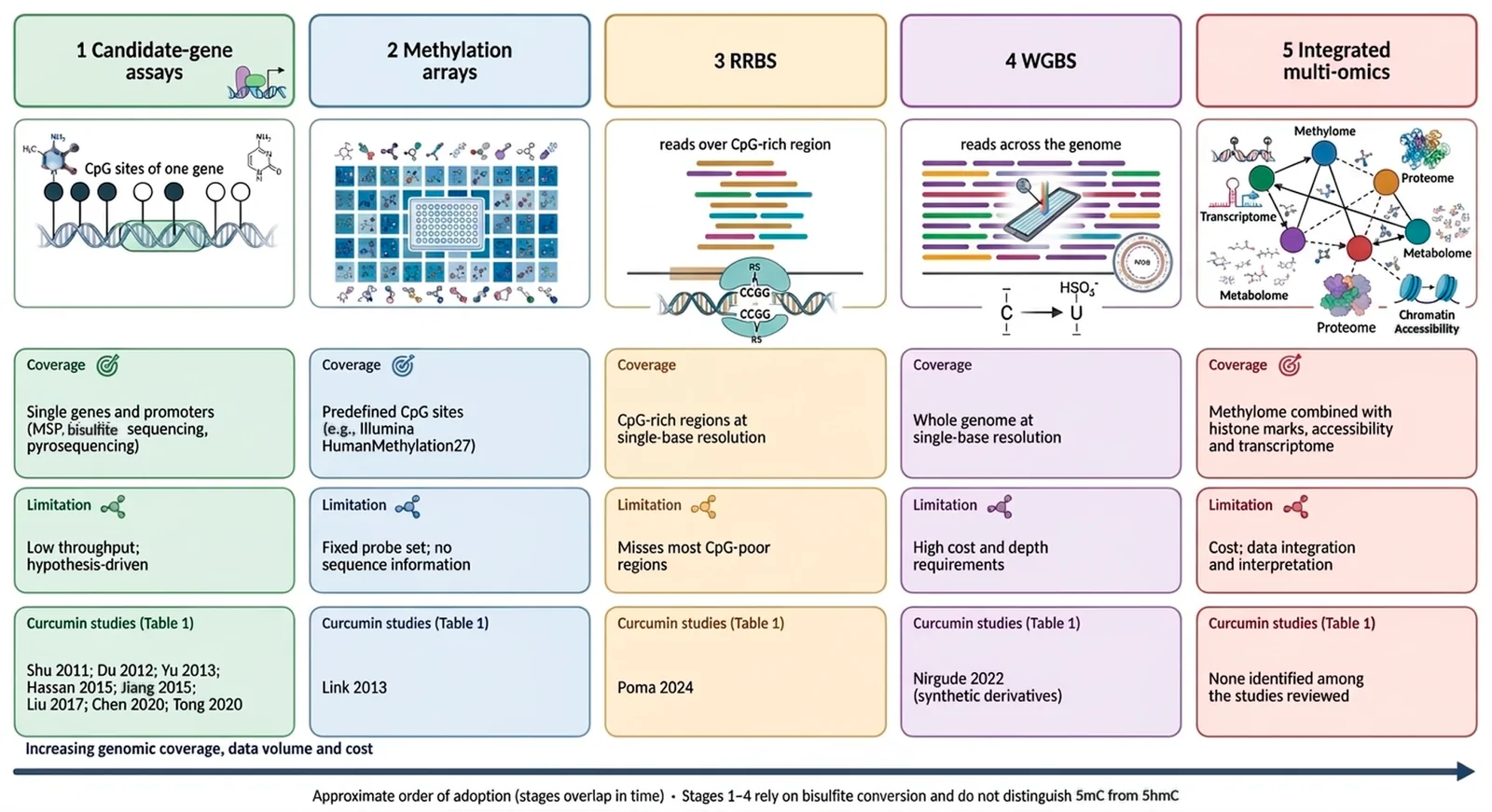
Methods used to study DNA methylation, from candidate-gene assays to integrated multi-omics (approximate order of adoption; stages overlap), with their coverage, limitations and the curcumin studies in Table 1 that used each approach [14,15,24,25,26,27,28,29,30,31,32].

The last decade has complicated that picture considerably. WGBS, RRBS, ChIP-seq, ATAC-seq, Hi-C, single-cell multi-omics are technologies that, taken together, are consistent with a more interconnected picture than a set of independent enzymes; gene regulation looks much more like the output of a densely wired network [5,6,20,21] (Figure 1).

DNA methylation is a good case in point. Promoter CpG island methylation is classically tied to gene silencing, but elsewhere (gene bodies, enhancers, CpG shores, repetitive regions), its effects on transcription, chromatin structure and genome stability differ and are sometimes opposite to those at promoters [10,22]. Global methylation levels alone are, therefore, of limited interpretive value and need to be considered together with locus-specific and transcriptional data [5,23].

Enhancers add a further dimension: active enhancers, marked by H3K27ac and open chromatin, contact promoters through three-dimensional interactions and are central to lineage decisions and tumor progression [33,34]. A compound that alters chromatin structure may, therefore, act at a distance from promoters. Accessibility is a further regulatory layer: methylation changes do not always alter transcription unless accessibility and transcription-factor occupancy also change [21,35]. Multi-omics studies show extensive crosstalk among these layers [6,20], and in cancer, resistance is increasingly viewed as a reversible adaptive epigenetic state [9,11].

In this context, natural compounds with broad, pleiotropic activity have regained interest. A classical epidrug is built to hit one enzyme class. Many phytochemicals do not work that way—they touch inflammatory signaling, oxidative stress, metabolism, transcription factors and several epigenetic regulators more or less at once. It seems unlikely that such effects trace back to inhibition of a single chromatin-modifying enzyme. A more plausible explanation is that they perturb the network as a whole, with the outcome depending on the state of the cell they happen to be acting on [3,4,36].

Curcumin is among the most extensively studied compounds of this kind. Its interactions with DNMTs, HDACs and various non-coding RNAs have been documented extensively (Table 1), but genome-wide data now suggest that these individual targets are pieces of something larger, not the whole story. Curcumin does not simply block DNA methylation or histone deacetylation; it appears to touch several epigenetic layers at once, and the resulting chromatin and transcriptional remodeling depends heavily on cellular context. That framing goes some way toward explaining why the literature on curcumin looks so heterogeneous—and, at times, contradictory—across different experimental systems.

On this basis, we propose, as a working hypothesis, that part of curcumin’s biological activity may be interpreted as epigenetic network rewiring. Rather than producing a uniform change across individual marks, curcumin appears to perturb interconnected circuits—DNA methylation, hydroxymethylation, histone modifications, chromatin accessibility, non-coding RNA networks—simultaneously. The resulting transcriptional response would then be expected to be locus-specific, cell-type-dependent, and heavily shaped by whatever epigenetic state the cell was in to begin with. This is the framework we use to interpret the evidence discussed in the sections that follow.

### Pharmacological Constraints on the Interpretation of Epigenetic Effects

Any interpretation of the epigenetic effects of curcumin must take its pharmacology into account. Curcumin has very low systemic bioavailability after oral administration because of poor aqueous solubility, chemical instability at physiological pH, and rapid glucuronidation and sulfation; in human studies, free curcumin is usually undetectable or present only at low nanomolar concentrations in plasma, even after high doses [37,38].

By contrast, most of the in vitro studies discussed in this review used concentrations in the micromolar range (Table 1), one to three orders of magnitude above plasma levels, although higher local concentrations may be reached in the gastrointestinal tract and with some formulations. Findings obtained at micromolar concentrations should, therefore, not be assumed to be achievable in patients.

In addition, curcumin is not a selective ligand. It is a reactive Michael acceptor, a redox-active phenolic compound and an iron chelator, and it is frequently identified as a promiscuous hit in biochemical assays, a behavior typical of pan-assay interference compounds [39,40]. These properties may generate reactive oxygen species and DNA damage at the concentrations used in vitro, and they can affect enzymes that depend on iron, 2-oxoglutarate or reactive thiol residues without implying a specific interaction. Changes in DNMT, HAT, HDAC or TET readouts after curcumin exposure may, therefore, be indirect consequences of redox stress, chelation or cytotoxicity rather than evidence of epigenetic targeting, and the term “epigenetic modulator” is used in this review in an operational sense. Native curcumin, synthetic curcuminoids and nano- or lipid-based formulations differ in stability, cellular uptake and potency, and the studies discussed below are not interchangeable. For this reason, Table 1 reports, wherever it could be verified, the compound, cell model, concentration, exposure time and assay used in each study.

## 3. Genome-Wide Remodeling of the DNA Methylation Landscape

### 3.1. Evolution of DNA Methylation Concepts: From Promoter-Centric Regulation to Methylome Architecture

DNA methylation is among the most extensively studied epigenetic modifications in eukaryotic cells, long regarded as the principal mechanism behind stable transcriptional repression. In mammals, it consists mainly of a methyl group attached to the fifth carbon of cytosine within CpG dinucleotides, producing 5-methylcytosine (5mC). DNA methyltransferases (DNMTs) carry out this reaction, and their coordinated activity establishes and maintains methylation patterns throughout embryonic development, tissue differentiation, and adult tissue homeostasis [5,18,19].

Early mechanistic models focused almost entirely on promoter-associated CpG islands. Methylation of CpG-dense promoter regions blocks transcription-factor binding and recruits methyl-CpG-binding proteins and histone-modifying complexes, leading to chromatin condensation and gene silencing [19,41]. This model became the default framework in molecular biology and cancer epigenetics, since it offered a straightforward explanation for the epigenetic inactivation of tumor suppressor genes found across many human cancers [19].

High-throughput sequencing changed that picture [42,43]. Large consortium efforts—the Human Epigenome Project, ENCODE, the NIH Roadmap Epigenomics Consortium—showed that DNA methylation is not uniformly distributed across the genome but organized into patterns that vary by genomic location, developmental stage, and cell identity [44,45,46]. The mammalian methylome turned out to be a spatially arranged regulatory system, not a collection of independently methylated promoters.

Genomic context largely determines the effect of a methylation mark: CpG shores, shelves, open-sea regions, gene bodies, repeats and distal regulatory elements act through different mechanisms [22,46]. Methylation changes outside CpG islands often correlate better with tissue-specific expression than those within promoter islands [47]. Gene-body methylation often correlates positively with expression and has been linked to elongation and alternative splicing [48]; enhancer methylation modulates accessibility and transcription-factor occupancy and is central to lineage specification and cancer [33,49,50].

A further conceptual shift concerns how methylation relates to chromatin organization more broadly. It is no longer treated as an isolated regulatory layer but as one component of a network that continually interacts with histone modifications, nucleosome positioning, chromatin accessibility, three-dimensional genome architecture, and non-coding RNAs [5,6]. The same methylation change can, therefore, produce different biological outcomes depending on the surrounding chromatin environment and the transcriptional state of that region [10,35,51,52].

All of this changes how the biological effects of epigenetically active compounds should be interpreted. Older pharmacological studies tended to score a compound by whether it raised or lowered global methylation or reactivated a small set of silenced genes. Those measures remain useful, but they cannot distinguish methylome reorganization from effects confined to a handful of loci. Current epigenomic work, therefore, increasingly relies on genome-wide detection of differentially methylated regions (DMRs), paired with transcriptomic data and, where possible, chromatin-architecture information, to infer actual biological function [20,21].

The central question about curcumin has shifted accordingly. Early work asked whether curcumin inhibits DNMT activity and demethylates the promoters of tumor suppressor genes—a productive line of inquiry but one framed before genome-wide epigenomics existed. The more relevant question today is whether curcumin reshapes methylation selectively, depending on genomic context, cell identity, and disease state. Answering it requires moving past promoter-focused studies toward integrated, methylome-wide approaches capable of capturing that complexity.

The remainder of this section follows that trajectory. We begin with the classical evidence for curcumin’s effect on DNMTs, then turn to what genome-wide methylation studies have added to—and complicated—that picture. The available data argue against describing curcumin as a simple DNA-demethylating agent and are compatible with selective, context-dependent changes in methylation, although the number of genome-wide studies is small.

### 3.2. DNA Methyltransferases and the Early Epigenetic Evidence Concerning Curcumin

Curcumin’s epigenetic activity first surfaced in studies of DNA methyltransferases rather than in genome-wide methylation data. This distinction is relevant because changes in DNMT activity or expression do not, in themselves, determine the direction of the resulting methylation changes, magnitude, or genomic distribution of any resulting methylation change. Still, these early studies gave curcumin a plausible mechanistic route into epigenetic regulation.

DNMT1 mainly maintains existing methylation patterns during DNA replication, while DNMT3A and DNMT3B establish new marks, though this division of labor is not absolute, since maintenance and de novo methylation overlap and are determined by chromatin context, replication timing, and protein interactions [22,48]. A drop in DNMT1 can, therefore, cause passive methylation loss in dividing cells, and changes in DNMT3A or DNMT3B can alter methylation acquisition at specific loci. Neither mechanism, on its own, produces genome-wide demethylation.

**Figure 2:**
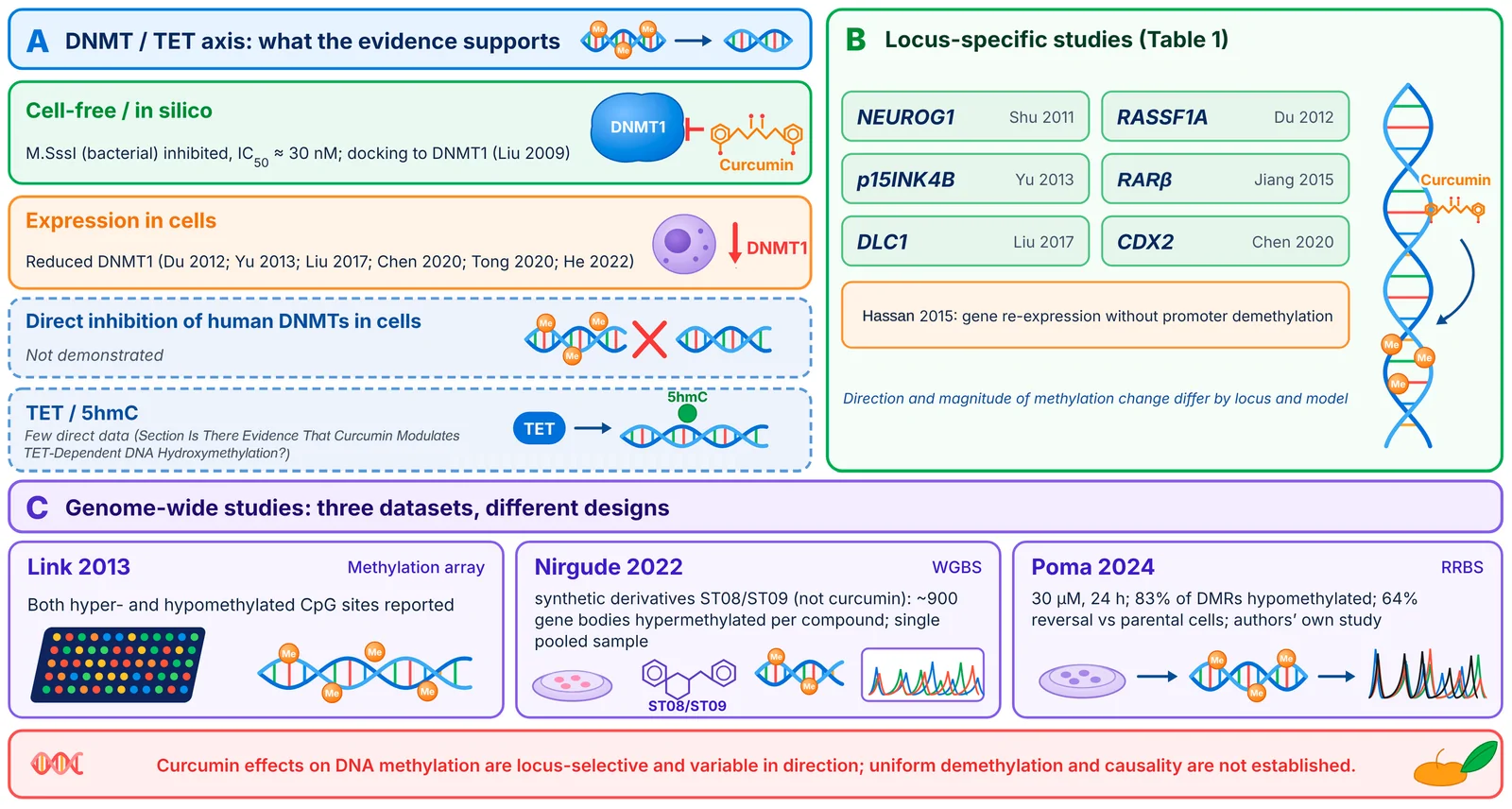
Evidence on curcumin and DNA methylation. (**A**) Strength of evidence for the DNMT/TET axis. (**B**) Locus-specific studies, see Table 1 for more details. (**C**) Genome-wide studies. Effects are locus-selective and vary in direction; uniform demethylation and causality are not established [14,15,24,25,26,27,29,30,31,32,53,54,55].

One of the earliest and most cited studies reported that curcumin inhibits CpG methyltransferase activity in vitro and lowers global DNA methylation in a leukemia cell model [53]. Molecular docking suggested curcumin could bind the catalytic pocket of DNMT1, potentially blocking the catalytic thiolate of Cys1226. The experimental part used the bacterial CpG methyltransferase M.SssI, which curcumin inhibited at a reported nanomolar IC50; in a leukemia-cell model, curcumin treatment also reduced global DNA methylation [53] (Figure 2). This gave hypomethylation observed elsewhere a plausible biochemical mechanism.

The evidence needs qualifying, though. The inhibition assay used M.SssI, a bacterial enzyme, not purified human DNMT1—the covalent interaction with the human enzyme rests mainly on modeling. M.SssI inhibition is a reasonable proxy for CpG methyltransferase activity, but it is not a direct demonstration that human DNMT1 is inhibited. And a global methylation readout cannot say which genomic regions changed, or whether the change indicates a primary enzymatic effect versus altered proliferation, cell-cycle shifts, or cellular stress. Read narrowly, this study is solid proof of principle; read as proof that curcumin directly and selectively inhibits human DNMT1 in cells, it overreaches.

Locus-specific follow-up work linked curcumin to demethylation and re-expression of silenced genes. In LNCaP prostate cancer cells, curcumin lowered methylation across the first 14 CpG sites examined in the NEUROG1 promoter’s CpG island and restored NEUROG1 expression [24], together with reduced DNMT1 expression and altered local chromatin marks. That the study tied together promoter methylation, gene expression, and chromatin state in one system is its strength; its narrow focus on a single locus is its limit—it cannot say whether NEUROG1 was a preferential target or one instance of a wider response (Figure 2).

A comparable result emerged for RASSF1A in breast cancer cells [25]. Curcumin treatment of MCF-7 cells reduced methylation-associated repression and restored RASSF1A expression, accompanied by lower nuclear methyltransferase activity and reduced DNMT1 mRNA and protein. Mechanistically, this was linked to a disrupted NF-κB/Sp1-dependent regulation of the DNMT1 promoter [25], evidence that curcumin can act on DNMT1 indirectly, through transcription-factor signaling, rather than only through direct enzyme inhibition.

Yu and colleagues pursued that indirect route further in acute myeloid leukemia, displaying reduced DNMT1 expression across AML cell lines, primary AML cells ex vivo, and a murine model, alongside decreased expression and promoter binding of NF-κB p65 and Sp1 [26]. In that study (10–20 µM, 24–72 h in vitro; 100 mg/kg i.p. in mice), the DNMT1 decline was accompanied by hypomethylation (about 40%, measured by LC-MS/MS) of the p15INK4B promoter and re-expression of p15INK4B and by reduced proliferation and increased apoptosis in the leukemia cells; methylation of other genes and global methylation were not reported [26] (Figure 2). Spanning in vitro, ex vivo, and in vivo systems, this constitutes a broader dataset than the candidate-locus work before it, but the link between lower DNMT1, hypomethylation, and antileukemic activity remains largely associative. Curcumin touches many signaling and stress pathways that can affect cell survival independent of methylation, and this study does not establish whether demethylation preceded transcriptional reactivation and apoptosis or occurred alongside them as part of a wider cellular response.

Link and colleagues took a different approach, moving from single genes to array-based methylation profiling in colorectal cancer cell lines (HCT116, RKO, HT29), using 5-aza-2′-deoxycytidine as a reference hypomethylating agent [27]. Where 5-aza-2′-deoxycytidine drove broad hypomethylation, curcumin did not—it produced both hyper- and hypomethylation at a limited set of loci, concentrated at CpGs with intermediate baseline methylation (Figure 2).

A separate line of leukemia work complicated the hypomethylation narrative further. Hassan and colleagues compared native curcumin with dimethoxycurcumin, finding that both altered DNMT isoform expression and reactivated some silenced genes, yet quantitative pyrosequencing of the p15 and CDH1 promoters showed no meaningful reversal of methylation under the tested conditions (1–2 µM for 72–168 h; 5–10 µM for 72–120 h in CEM cells) [28]. Genes were re-expressed without corresponding promoter demethylation.

That result is worth pausing on. It shows that reactivating a methylation-silenced gene is not, by itself, evidence of demethylation: transcription can be restored through histone acetylation, transcription-factor recruitment, nucleosome repositioning, or other chromatin mechanisms even while promoter methylation stays largely unchanged. Hassan et al. also emphasized that curcumin and dimethoxycurcumin are not interchangeable; dimethoxycurcumin is more metabolically stable, and differing intracellular exposure may shape both epigenetic and cytotoxic outcomes.

Taken together, studies finding demethylation and studies finding little or none are not simply contradictory. They differ in cell lineage, baseline methylation, proliferative state, compound concentration, exposure time, and analytical method, and in what they actually measured, whether global methylation, a single promoter, or DNMT expression alone. These are related but not identical questions.

Additional candidate-locus studies confirmed the hypothesis that curcumin can lower methylation at select tumor-suppressive or differentiation-related genes: RARβ re-expression via promoter demethylation in cancer cells [29]; demethylation and re-expression of DLC1 in breast cancer models, alongside growth inhibition [30]; and, in colorectal SW480 cells, reduced DNMT1/DNMT3A expression together with CDX2 promoter demethylation, linked to shifts in CDX2/Wnt/β-catenin signaling and EMT markers [31] (Figure 2).

These data are biologically plausible but methodologically constrained—mostly single cell lines, short exposures, one locus at a time. Curcumin concentrations were often chosen from cytotoxicity assays without measuring intracellular drug levels, and causal claims typically rested on parallel changes in DNMT expression, promoter methylation, and gene expression rather than on rescue experiments or targeted epigenetic editing. Locus-specific demethylation observed at the population level could also reflect selection of a subpopulation rather than a uniform variation within individual cells (Figure 2).

A 2020 study in gastric cancer cells proposed yet another route: curcumin-induced DNA damage, growth arrest, and activation of DNA-damage-response pathways, accompanied by reduced DNMT1 expression and re-expression of demethylation-associated genes [32]. Since DNA repair, replication, and methylation maintenance are mechanistically intertwined, this is a reasonable mechanism, but it also blurs curcumin’s status as a selective epigenetic agent. At DNA-damaging concentrations, the observed methylation changes could just as plausibly reflect replication stress or repair-associated chromatin remodeling.

More recent work continues to find context-specific DNMT modulation. Curcumin sensitized non-small-cell lung cancer cells to crizotinib, an effect linked to restored miR-142-5p expression and reduced DNMT1/DNMT3A/DNMT3B [54]. This supports crosstalk between methylation and non-coding RNA networks without establishing DNMT downregulation as the sole driver of sensitization; more plausibly, curcumin perturbs several related regulatory systems at once.

Four conclusions follow from this body of DNMT- and candidate-gene work. Curcumin can lower DNMT1 expression across multiple models, usually indirectly via NF-κB and Sp1. It is associated with demethylation and re-expression at certain genes, though the effect varies by gene and model. Gene reactivation can occur without detectable promoter demethylation, pointing to methylation-independent chromatin mechanisms. And nothing here supports treating curcumin as a uniform, universally active demethylating agent.

DNMT modulation is, thus, one entry point into a larger epigenetic response, shaped by baseline methylation, cell division, chromatin accessibility, transcription-factor signaling, and the rest of the epigenetic machinery. That is the case for moving from candidate-gene evidence to a direct look at curcumin-induced methylome architecture.

### 3.3. Progressing from Candidate Loci to Genome-Wide Methylome Analysis

Moving from single-gene studies to genome-wide profiling represented a real advance in understanding curcumin’s epigenetic effects. Locus-specific work showed that curcumin can affect DNMT expression, promoter methylation, and gene transcription, but it could not determine whether the examined loci reflected a broader methylome response or were simply chosen for known biological relevance. For a pleiotropic compound, that distinction is not academic: methylation loss at one promoter could exist together with methylation gain elsewhere, with no net genome-wide change, or with delayed changes appearing only after prolonged adaptation. Genome-wide analysis is the only way to tell general demethylation apart from selective redistribution.

#### 3.3.1. The Initial Evidence from Array-Based Methylome Studies

Link and co-workers ran the first systematic methylome-level study of native curcumin in cancer cells [27] (Figure 2), using three colorectal lines with different genetic and epigenetic backgrounds: HCT116 (microsatellite instability), RKO (CpG island methylator phenotype), and HT29 (microsatellite stable). Cells received subtoxic or antiproliferative curcumin doses—7.5 μM for HCT116, 10 μM for RKO and HT29—under two exposure regimes: 6 days and 240 days. Both concentrations are in the micromolar range and exceed the plasma levels reachable after oral administration (see Section Pharmacological Constraints on the Interpretation of Epigenetic Effects).

Methylation was measured with the Illumina HumanMethylation27 BeadChip, covering 27,578 CpG sites across more than 14,000 genes. Called “genome-wide” at the time, the array was in practice heavily biased toward promoter- and CpG-island-associated CpGs, leaving distal enhancers, intergenic regions, repetitive sequences, partially methylated domains, and much of the gene body under-sampled—a coverage limitation discussed in Section 3.3.4 (see “What counts as ‘genome-wide’ evidence?”).

Including 5-aza-2′-deoxycytidine and trichostatin A as controls strengthened the study considerably [27]. 5-aza-2′-deoxycytidine produced broad hypomethylation with a clear shift in mean methylation values; trichostatin A barely moved methylation at all. Curcumin looked like neither. Both the array distributions and LINE-1 pyrosequencing showed no general loss of methylation, only changes at a limited set of CpGs, with overall methylation density essentially unchanged [27]. Curcumin and nucleoside DNMT inhibitors were, in other words, doing mechanistically different things, and neither the DNMT-expression changes nor the promoter-specific demethylation could be extrapolated to the genome as a whole.

Comparing short- and long-term treatment was especially informative. Thus, 6 days of exposure produced only modest methylation changes across all three lines; 240 days produced far more differentially methylated CpGs [27]. Applying a Δβ threshold of 0.1 with independent replicate arrays, the researchers identified 1436 reproducibly altered sites in HCT116, 814 in RKO, and 3051 in HT29 after long-term treatment (Figure 2).

More sites changed, but average methylation barely moved—a redistribution, not an erasure. Gains and losses both occurred, concentrated at CpGs with intermediate baseline methylation; fully methylated and fully unmethylated CpGs were largely unresponsive [27]. That pattern fits with curcumin acting preferentially on epigenetically dynamic or metastable regions. Intermediate β-values can reflect cellular heterogeneity, allele-specific methylation, or regulatory regions whose methylation state is not fixed—sites that may be more susceptible toward shifts in transcription-factor occupancy, chromatin accessibility, replication dynamics, or DNMT recruitment. Bulk-array data cannot, however, distinguish between these possibilities. It should also be noted that intermediate β-values are subject to higher technical variance on this array platform and that the Δβ > 0.1 threshold with a limited number of replicates may include sites whose change reflects measurement noise; the biological relevance of individual sites is, therefore, uncertain.

The long exposure window adds its own interpretive complication. Stable methylation differences after 240 days need not reflect a direct molecular effect of curcumin; repeated transcriptional disruption, metabolic adaptation, subclone selection, and shifting proliferation rates over many cell generations could all produce the same signature. Notably, Link and colleagues found gene-expression changes already after short-term exposure, while short-term methylation patterns resembled untreated controls more than they resembled the long-term treated pattern [27]. That ordering hints that at least some methylation changes are downstream of transcriptional reprogramming rather than its cause, worth keeping in mind generally: a compound can act quickly on transcription factors or signaling pathways, with methylation arriving later to stabilize a transcriptional state already in place.

#### 3.3.2. Cell Identity Shapes the Methylation Changes Induced by Curcumin

The three colorectal models responded very differently. Only 68 hypomethylated sites were shared across all three [27]—a small overlap that says a lot about how much the starting genetic and epigenetic state shapes the response. HCT116, RKO, and HT29 serve as distinct colorectal cancer epigenotypes: RKO carries extensive CpG-island methylation, while HCT116 and HT29 differ in mutation pattern and baseline methylation structure. Curcumin was, in effect, acting on three separate regulatory backgrounds rather than a fixed, universal set of targets.

These findings provide preliminary, albeit limited, support for the argument developed in this section. If curcumin worked purely through indiscriminate catalytic inhibition of DNMT1, more uniform changes would be expected, especially in heavily methylated regions. Instead, the response was cell-line-specific, possibly shaped by baseline DNMT/TET expression, proliferation rate, chromatin accessibility, transcription-factor availability, methyl-donor supply, and the pre-existing epigenetic state of individual loci.

#### 3.3.3. Integrating Methylation with Transcription

Link and colleagues [27] also combined the methylation data with gene-expression microarrays, flagging genes with at least 10% methylation change and 1.5-fold expression change: 235 in HCT116, 108 in RKO, 543 in HT29. These genes clustered around cancer biology, inflammation, metabolism, molecular transport, signaling, and cell-cycle regulation. Combining the two datasets was methodologically valuable, and it exposed a real limit at the same time. Only some of the methylation changes showed the expected inverse relationship with expression—more methylation, less expression, or the reverse. Others showed no associated expression change, or patterns that are not consistent with simple promoter-repression model at all. Several explanations are plausible: a single array probe could not capture a whole CpG island’s regulatory behavior; methylation may only matter within specific transcription-factor motifs or chromatin contexts; some genes may need coordinated histone and chromatin accessibility changes before a methylation change becomes visible in expression; and some expression changes may be driven mainly by signaling, independent of methylation altogether. The wider lesson—correlation between methylation and expression is not causation, and only time-resolved or targeted-manipulation experiments can establish direction—resurfaces in the WGBS and RRBS studies discussed later.

#### 3.3.4. From Arrays to Sequencing

Array profiling improved on candidate-gene analysis, whereas sequencing-based methods extend this approach by offering coverage that is not restricted to predetermined targets. Bisulfite sequencing converts unmethylated cytosines to uracil while leaving 5mC untouched, giving single-base methylation resolution. Two sequencing approaches dominate curcuminoid research: reduced-representation bisulfite sequencing (RRBS) and whole-genome bisulfite sequencing (WGBS).

RRBS enriches CpG-dense regions through restriction digestion, size selection, and bisulfite sequencing [56,57]. It is more cost-effective than WGBS and gives single-nucleotide resolution well suited to CpG islands and promoters, but coverage depends on where restriction sites happen to fall and which fragments are selected, so large stretches of the genome, including many enhancers, intergenic regions, and repetitive sequences, are simply not sampled. RRBS is best thought of as a high-resolution map of CpG-rich territory, not a whole-genome assay.

WGBS, in principle, captures nearly every uniquely mappable cytosine, covering promoters, gene bodies, enhancers, open-sea CpGs, and partially methylated domains without any restriction-enzyme enrichment step [58]. That completeness comes at a cost: high sequencing depth, heavy computation, and continued sensitivity toward uneven coverage, mapping difficulty in repetitive regions, and weak statistical power when replication is thin.

One limitation applies to both methods equally. Standard bisulfite conversion cannot tell 5mC apart from 5-hydroxymethylcytosine (5hmC)—both read as “methylated.” Some of the methylation changes reported by the RRBS and WGBS studies discussed below may, therefore, partly reflect hydroxymethylation rather than methylation itself. This caveat applies to all studies discussed below and is treated in more detail in Section 4.

What counts as “genome-wide” evidence?

The term “genome-wide” is used loosely in the curcumin literature. The HumanMethylation27 array Link et al. used covered tens of thousands of CpGs across thousands of genes—reasonably called genome-scale relative to candidate-gene assays of its time [27]—but it still captured only a small, promoter-biased slice of the roughly 28 million CpG sites in the human genome. RRBS sharpens resolution while staying deliberately confined to CpG-dense regions. WGBS offers the broadest coverage, though its effective resolution still depends on sequencing depth, read quality, replicate number, and analytical thresholds.

Within the progression from candidate-gene assays to arrays, RRBS, WGBS and integrated multi-omics described above, curcumin research has so far reached the sequencing-based methods (RRBS and WGBS) in only a handful of studies. Nobody has yet combined native curcumin treatment with WGBS, hydroxymethylome profiling, chromatin-accessibility mapping, and histone-mark analysis in the same biological system. That gap matters, because methylation data alone cannot reconstruct the full epigenetic landscape.

#### 3.3.5. Conceptual Implications

The move from single-promoter studies to genome-wide profiling has changed how curcumin’s biological effects are read. Candidate-gene work tied curcumin to promoter demethylation and gene re-expression. Array analysis then showed those effects sitting alongside methylation gains, concentrated at specific loci, and varying by cell line and treatment duration [27]. Sequencing-based studies confirmed effects across promoters, CpG islands, and gene bodies, while presenting compound-specific, context-dependent signatures [14,15].

The data available do not resemble those expected from a conventional non-selective hypomethylating agent. Methylation did not change uniformly across the methylome; changes were concentrated at specific loci and were bidirectional in the array and WGBS studies but predominantly hypomethylating in the RRBS study. “Methylome remodeling” is the more accurate term for this. “DNA demethylation” still fits individual loci where a decrease has been directly measured but not the genome as a whole unless global, region-resolved data back it up.

### 3.4. Whole-Genome Bisulfite Sequencing and Curcuminoid-Induced Methylation

WGBS allows quantitative, single-base methylation analysis across nearly the whole genome, extending measurement well beyond promoter CpG islands into gene bodies, intergenic regions, repetitive regions, and distal regulatory sequences [58,59]. Candidate-gene approaches ask which genes become demethylated; WGBS asks how methylation redistributes across the entire genome—a direct test of whether locus-specific changes are isolated or part of something broader.

Very few studies have applied WGBS to curcuminoids. The most thorough comes from Nirgude and colleagues, who examined two synthetic curcumin derivatives, ST08 and ST09, in the triple-negative breast cancer line MDA-MB-231 [14].

Neither derivative is native curcumin; both were chemically modified for greater cytotoxic potency, so these data concern structurally related curcuminoids and cannot be extrapolated to curcumin itself.

The experimental design combined WGBS with RNA sequencing to test whether methylation changes tracked with transcriptional ones, a meaningful improvement over earlier work, since it treated methylation not as a standalone readout but alongside actual expression changes. That distinction matters because differential methylation, on its own, does not guarantee any functional effect on transcription.

Both derivatives produced many differentially methylated sites, and again, the pattern was mixed rather than uniformly hypomethylating—hypo- and hypermethylated sites appeared across genomic compartments [14] (synthetic derivatives, not native curcumin). This is in line with array- and RRBS-based work, which also reported bidirectional changes rather than a uniform global shift.

Several genes showed altered promoter-CpG-island methylation, including members of the HOX transcription-factor family, genes frequently dysregulated epigenetically as breast cancer progresses. That raises the possibility that curcuminoid exposure touches developmental transcriptional programs still epigenetically active in malignant cells, though promoter changes alone cannot distinguish primary targets from downstream effects of more extensive chromatin remodeling.

Gene-body methylation changes were more extensive—hundreds of genes affected after exposure to either derivative [14] (synthetic derivatives, not native curcumin). That matters because gene-body methylation is now understood as an active regulator of transcriptional elongation, alternative splicing, and transcript stability, not a passive byproduct of transcription [38]. Changes outside promoters should not be assumed to be biologically secondary.

RNA-seq revealed that, despite their structural similarity, ST08 and ST09 (synthetic derivatives, not native curcumin) produced different biological signatures: ST08 mainly affected extracellular-matrix-organization genes; ST09 mainly affected cell-cycle regulation [14]. Structurally similar compounds producing different methylation and transcriptional programs argue against a single-enzyme explanation like DNMT1 inhibition; small structural differences appear capable of redirecting much larger regulatory networks.

This study has real limits. It used one cell line, so reproducibility elsewhere—other breast cancer models, other tumor types—is unknown. Cells were treated for 48 h with 75 nM (ST08) or 50 nM (ST09), and the Methods describe a single pooled sample per condition without biological replicates. Bisulfite conversion, as noted above, cannot separate 5mC from 5hmC, so some of the signal may reflect hydroxymethylation. And, without matching chromatin accessibility, histone modification, or 3D genome data, the full epigenetic response cannot yet be reconstructed. Methylation data alone, however precisely mapped, also cannot demonstrate causality—the changes could precede transcriptional shifts, follow altered transcription-factor occupancy, or represent adaptation to extended exposure. Including RNA-seq helps here, but pinning down the actual sequence of events will need chromatin accessibility and histone data on top of it.

The WGBS data from this single study are not easily reconciled with a purely DNMT-centered model. The synthetic curcuminoids tested appear to produce bidirectional, context-specific methylation changes that vary with genomic region and compound structure. Because only two synthetic derivatives and one cell line were examined, confirmation with native curcumin and additional models is required before this can be generalized.

### 3.5. Reduced-Representation Bisulfite Sequencing and Context-Specific Remodeling

WGBS is still the most complete method for genome-wide cytosine mapping, but its cost, computing requirements, and depth requirements keep it out of reach for many exploratory studies, particularly ones involving multiple biological replicates. RRBS was built to work around that, enriching CpG-dense regions through restriction digestion, size selection, and bisulfite sequencing [56,57]. RRBS is not merely a cost-effective alternative to WGBS; it is a complementary method, well suited to promoters, CpG islands, and regulatory regions, while WGBS remains the better tool for repetitive sequences, intergenic regions, and CpG-poor stretches of the genome [56,57,58,59].

The only RRBS study of native curcumin identified in our search was performed by Poma and colleagues, working in doxorubicin-resistant MCF-7 breast cancer cells [15]. All three authors of the present review are co-authors of that study, and we, therefore, limited our discussion to the findings reported there (30 µM curcumin for 24 h, two biological replicates per condition). The model is worth noting in its own terms: multidrug resistance is increasingly understood as a dynamic state maintained by coordinated transcriptional and epigenetic adaptation, not fixed genetic change. Rather than treating naïve cancer cells, this study asked whether curcumin could alter an already-established resistant epigenetic state—a harder, more clinically relevant question. In this study, RRBS was combined with RT-qPCR of selected genes, measurement of P-gp protein and an NF-κB DNA-binding assay; unlike the WGBS study above, no genome-wide transcriptome was generated, so the link between methylation and expression is established only for a limited set of genes [15].

RRBS turned up many differentially methylated regions across promoter CpG islands, gene bodies, and other CpG-rich stretches. In contrast to the array and WGBS studies discussed above, the effect was predominantly demethylating: curcumin raised the fraction of demethylated CpGs from about 25% to 37%, and 83% of the differentially methylated regions (|Δ| > 25%, q < 0.01) were less methylated, with changes concentrated at specific loci (ribosomal DNA arrays, genes involved in ribosome biogenesis, Wnt signaling, chromatin regulators and non-coding RNAs). Comparison with the drug-sensitive parental cells showed that 64% of the demethylated regions had become hypermethylated upon acquisition of doxorubicin resistance. The RRBS data, therefore, point to locus-selective rather than genome-wide demethylation, but the direction differs from that reported in the array and WGBS studies; dose, exposure time, compound and cellular background may all contribute, and none of these variables was tested systematically [15]. Methylation changes were examined together with the expression of selected genes by RT-qPCR; notably, the ABCB1 (P-gp) promoter itself showed no methylation change at 24 h, although P-gp protein was strongly reduced, indicating that this effect did not involve ABCB1 methylation [15]. These observations suggest that DNA methylation is one component of a larger regulatory system rather than a standalone determinant of transcription.

The resistant-cell model also points toward a connection between epigenetic remodeling and phenotypic plasticity. If drug resistance is a reversible, epigenetically maintained state, then the co-occurrence of methylome changes with transcriptional and phenotypic changes is compatible with effects beyond cytotoxicity, but the study does not demonstrate that the methylation changes cause the phenotypic shift. The comparison with the sensitive parental line indicates partial reversal of resistance-associated methylation at these loci, but it does not show that the resistant epigenome as a whole was restored, and the causal contribution of the methylation changes to the reversal of the phenotype was not tested (single concentration and time point, two biological replicates). This is consistent with, though not proof of, the more general idea that epigenetic therapies reprogram cell states rather than simply reversing individual molecular lesions [60,61].

The same limitations that apply to RRBS generally apply here. Coverage stays confined to CpG-enriched regions, so changes in distant enhancers, repetitive elements, or CpG-poor intergenic regions go undetected. Bisulfite conversion still cannot separate the 5mC from the 5hmC signal. And reconstructing the full sequence of molecular events behind curcumin’s effects will need more than methylation and transcription data—chromatin accessibility, histone modifications, and single-cell epigenomics all remain missing pieces.

The three genome-scale datasets do not give a uniform picture. The array study in colorectal lines reported bidirectional changes without global loss of methylation, the WGBS study of synthetic derivatives reported mixed changes (with hypermethylation of about 900 gene bodies per compound), and the RRBS study of native curcumin reported a predominantly demethylating effect (83% of DMRs). What they share is that changes were locus-selective rather than genome-wide, and none supports a uniform genome-wide demethylation. The direction of the changes, however, differs between systems, compounds, doses and exposure times (Table 1), and none of the findings has been independently replicated; one of the studies is from our group. These data are, therefore, preliminary and do not by themselves establish a general property of curcumin.

## 4. DNA Hydroxymethylation: Beyond the Limits of Conventional DNA Methylation Analysis

Although genome-wide DNA methylation profiling procedures, including array-based methylation platforms, reduced-representation bisulfite sequencing (RRBS), and conventional whole-genome bisulfite sequencing (WGBS), have substantially improved the understanding of curcumin-induced epigenetic remodeling, an important methodological limitation occurs. All these methods rely on sodium bisulfite conversion, a chemical reaction that efficiently discriminates unmethylated cytosines from methylated cytosines but cannot distinguish 5-methylcytosine (5mC) from 5-hydroxymethylcytosine (5hmC) [14,59,62,63,64]. Consequently, current methylation profiles capture a mere combined signal. This failure to separate the two marks complicates how we interpret epigenetic responses driven by natural agents like curcumin. This is a limitation, particularly since 5hmC is now recognized not as a minor, fleeting intermediate of DNA demethylation but as an independent regulatory mark governing clear biological tasks.

For decades, investigators treated DNA methylation as a rigid, mostly static epigenetic mark, and demethylation happened almost exclusively through passive dilution when DNA strands replicated. The discovery of the Ten-Eleven Translocation (TET) enzyme family challenged this assumption: TET1, followed by TET2 and TET3, catalyzes the iterative oxidation of 5mC to 5hmC, 5-formylcytosine (5fC) and 5-carboxylcytosine (5caC), the latter two being excised by thymine DNA glycosylase (TDG) and replaced with unmodified cytosine through base excision repair (BER), establishing the molecular basis of active DNA demethylation and introducing a completely new level of epigenetic regulation [65,66]. Ito and co-workers subsequently showed that TET proteins are required for efficient conversion of 5mC to 5hmC during embryonic development and contribute to embryonic stem-cell self-renewal and lineage specification [67]. DNA methylation is, therefore, a dynamic mark that can be modified in response to developmental, metabolic or environmental signals.

Subsequent studies demonstrated that 5hmC possesses its own genomic distribution and biological significance rather than representing only a transient intermediate in cytosine demethylation [68]. It has been shown that hydroxymethylation is preferentially enriched within gene bodies, active enhancers and lineage-specific regulatory elements [68,69], where it is frequently associated with transcriptionally active chromatin and increased cellular plasticity. Conversely, in promoter-associated regions, where DNA methylation generally correlates with transcriptional repression, 5hmC is often linked instead to transcriptional competence and dynamic regulation of gene expression [70]. Also, it has been shown that tissue-specific hydroxymethylation patterns are considerably more variable than DNA methylation profiles [68,71], thus suggesting that 5hmC contributes to the establishment and maintenance of cellular identity.

Altered TET expression, impaired catalytic activity and global loss of 5hmC have been reported in numerous human malignancies, including leukemia, glioma, melanoma, colorectal carcinoma and breast cancer [72,73,74]. In many cases, decreased hydroxymethylation correlates with tumor progression, increased cellular dedifferentiation and poor clinical outcome, supporting the concept that disruption of active DNA demethylation contributes to malignant transformation. TET activity is tightly regulated by intracellular metabolism. Because these enzymes require Fe(II), molecular oxygen and α-ketoglutarate as enzymatic cofactors, alterations in mitochondrial metabolism, oxidative stress and oncometabolite accumulation directly influence hydroxymethylation dynamics. By contrast, metabolites such as succinate, fumarate and the oncometabolite 2-hydroxyglutarate competitively inhibit TET activity, thereby linking cellular metabolism with epigenetic regulation [72].

These findings are relevant to the interpretation of curcumin studies. Beyond its reported effects on DNA methyltransferases, curcumin modulates multiple signaling pathways involved in oxidative stress, mitochondrial function, inflammatory responses and cellular metabolism [2,4,28]. Because all these processes influence TET activity either directly or indirectly, it is conceivable that part of the epigenetic effects of curcumin involves the TET–5hmC axis; this remains a hypothesis, as no study has directly demonstrated it.

### Is There Evidence That Curcumin Modulates TET-Dependent DNA Hydroxymethylation

Despite an expanding body of evidence demonstrating that curcumin influences DNA methylation, only a few studies have directly investigated whether these epigenetic effects involve modulation of the TET family of dioxygenases or alterations in DNA hydroxymethylation. Indeed, while inhibition of DNA methyltransferases has been extensively explored, the possibility that curcumin also regulates the active arm of DNA methylation dynamics has received considerably less attention. Current insights suggest that any relationship linking curcumin to TET performance is likely indirect, rather than arising from direct enzymatic activation. TET proteins remain highly sensitive to local metabolic conditions. Their catalytic drive depends completely on molecular oxygen, ferrous iron (Fe^2+^), and α-ketoglutarate; conversely, oxidative stress, iron oxidation, and the accumulation of cellular metabolites like succinate, fumarate, or 2-hydroxyglutarate quickly suppress their functional capacity [67,75]. Consequently, external compounds that disrupt cellular redox homeostasis or mitochondrial pathways can secondarily alter DNA hydroxymethylation patterns, without ever directly touching the catalytic pocket of TET enzymes. Curcumin is well known to modulate intracellular reactive oxygen species, mitochondrial function and multiple inflammatory signaling pathways, including NF-κB, PI3K/AKT and MAPK cascades [2,4,28]. In addition, curcumin chelates iron [76], which could in principle reduce the Fe(II) available to the TET catalytic site. These routes are speculative: none of the studies discussed in this review measured TET activity, 5hmC, or α-ketoglutarate/succinate levels after curcumin exposure, and whether iron chelation or redox effects occur at relevant intracellular concentrations is unknown (see Section Pharmacological Constraints on the Interpretation of Epigenetic Effects). Even so, direct experimental evidence demonstrating that curcumin increases global or locus-specific 5hmC levels through TET activation remains scarce [77,78,79]. An additional layer of complexity arises from the reciprocal relationship between DNA methyltransferases and TET enzymes. DNA methylation homeostasis is maintained through the dynamic balance between methylation and oxidation rather than by the activity of either enzyme family alone. As a result, a reduction in DNMT expression does not necessarily result in net DNA demethylation if TET activity is simultaneously reduced. Because bisulfite-based methods do not distinguish 5mC from 5hmC (Section 3.3.4), a change in apparent methylation cannot be attributed to either arm of this balance without oxBS-seq or TAB-seq [67,68,69,70,71,72,73,74,75]. Loss of 5hmC is a common feature of many tumors (see above), which makes the question relevant, but no data currently indicate that curcumin restores it. At present, however, the available evidence does not allow definitive conclusions. The field is limited by the scarcity of studies simultaneously quantifying 5mC, 5hmC and TET activity in the same experimental model. Addressing this limitation will require integrated experimental strategies combining oxidative bisulfite sequencing or TET-assisted bisulfite sequencing with transcriptomics, chromatin accessibility profiling and functional validation. Such multidimensional approaches will be essential to determine whether DNA hydroxymethylation represents a true driver of curcumin’s pharmacology or merely an epiphenomenon.

The recognition that DNA methylation and DNA hydroxymethylation represent functionally distinct yet interconnected epigenetic modifications has important implications for the design of future studies investigating curcumin activity. It should be emphasized that the proposed link between curcumin and TET activity, mediated by metabolic and redox-dependent pathways, remains hypothetical. To date, it lacks direct experimental support, such as measurements of TET enzymatic activity or 5-hydroxymethylcytosine levels following curcumin exposure, and it should not be regarded as an established mechanism. Much of the current literature has relied on experimental approaches that quantify global methylation or conventional bisulfite-derived methylomes without distinguishing individual cytosine derivatives. Although these studies have significantly expanded our understanding of curcumin-induced epigenetic remodeling, they are intrinsically unable to establish whether the observed changes originate from altered maintenance methylation, active demethylation or simultaneous modulation of both processes. As a result, future investigations should move beyond the analysis of total methylation levels toward integrated characterization of the entire cytosine modification landscape.

One obvious priority will be the routine incorporation of hydroxymethylome profiling into experimental workflows. Oxidative bisulfite sequencing (oxBS-seq), TET-assisted bisulfite sequencing (TAB-seq) and, more recently, long-read sequencing technologies based on nanopore or single-molecule real-time platforms now permit direct discrimination of modified cytosines with progressively increasing resolution [62,63,80]. These approaches overcome one of the principal limitations of conventional bisulfite sequencing and would allow investigators to determine whether loci previously classified as differentially methylated actually undergo hydroxymethylation, thereby providing a considerably more accurate interpretation of cytosine dynamics [35,67,81,82,83,84,85].

## 5. Histone Landscape Remodeling: From Enzymatic Targets to Chromatin Reorganization

### 5.1. Curcumin Remodels Histone Regulatory Networks Rather than Individual Histone Marks

Following the growing recognition that DNA methylation alone cannot fully explain the epigenetic effects of curcumin, increasing attention has shifted toward histone modifications as an additional regulatory layer governing chromatin architecture, transcriptional regulation and cellular plasticity [4,5,6,9,23,28,35]. Together with DNA methylation and chromatin accessibility, post-translational histone modifications orchestrate the dynamic organization of chromatin, determining whether specific genomic regions remain transcriptionally permissive or become epigenetically silenced [33,35]. As a result, understanding the epigenetic activity of curcumin requires moving beyond the analysis of DNA methylation alone toward a broader interpretation of chromatin regulation as an integrated and highly interconnected process.

**Figure 3:**
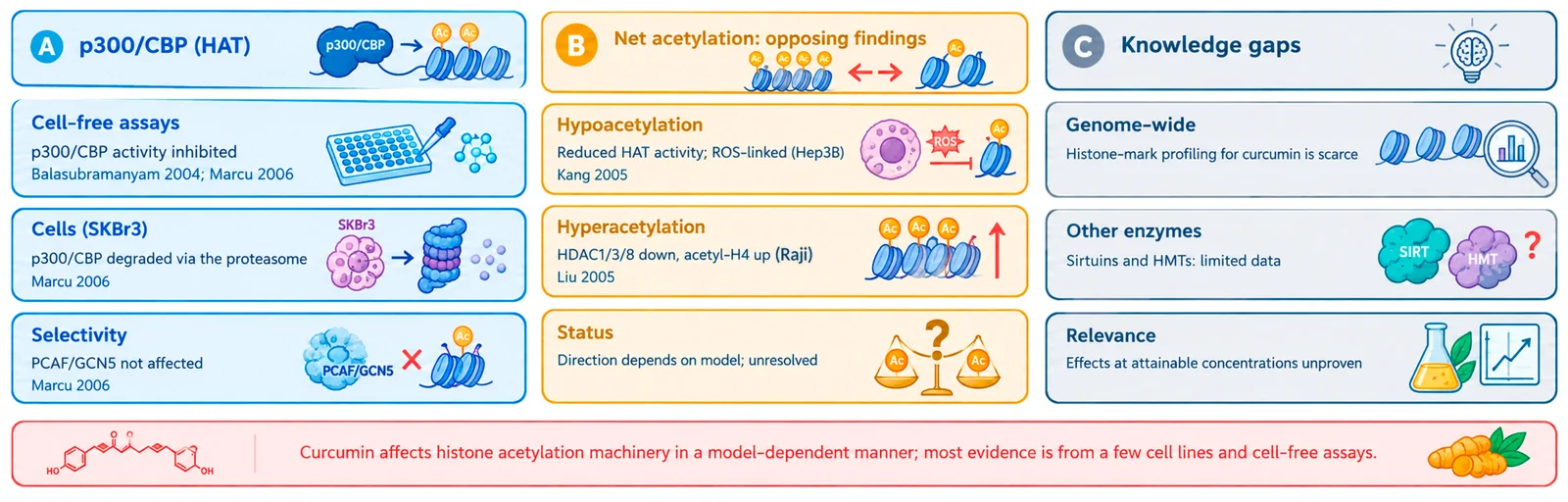
Curcumin and histone acetylation. (**A**) Effects on p300/CBP. (**B**) Opposing findings on net histone acetylation with the direction of the response that remains unresolved and appears to depend on the experimental model. (**C**) Knowledge gaps including limited genome-wide histone-mark profiling, insufficient evidence on sirtuins and HMTs, and uncertain physiological relevance [86,88,89,90].

Initial investigations into the epigenetic activity of curcumin were largely driven by the discovery that the compound inhibits the histone acetyltransferase (HAT) activity of p300/CBP in cell-free assays [86,87,88]. At the time, this observation represented a major conceptual advance, providing the first direct evidence that curcumin could target chromatin-modifying enzymes in addition to transcription factors and inflammatory signaling pathways (Figure 3). These inhibitory effects were obtained largely in cell-free assays or at micromolar concentrations, and curcumin is a reactive Michael acceptor (Section Pharmacological Constraints on the Interpretation of Epigenetic Effects); selective engagement of p300/CBP in cells at attainable concentrations has not been demonstrated.

However, the progressive accumulation of genome-wide transcriptomic and epigenomic data has substantially refined this interpretation. Rather than acting through modulation of a single histone acetyltransferase, curcumin appears to influence multiple chromatin regulatory pathways operating simultaneously and in a highly context-dependent manner [4,14,15]. In parallel with the evolution observed for DNA methylation, the field has progressively shifted from an enzyme-centered view toward a systems-level perspective in which histone modifications are considered components of complex regulatory networks interacting with DNA methylation, chromatin accessibility, enhancer activity and non-coding RNAs [6,9,35]. This conceptual transition is particularly important because alterations in histone acetylation rarely occur as isolated molecular events but are typically accompanied by coordinated remodeling of several epigenetic layers controlling gene expression.

Histone modifications themselves constitute a highly dynamic regulatory system. Acetylation, methylation, phosphorylation, ubiquitination and numerous additional post-translational modifications collectively regulate nucleosome stability, higher-order chromatin organization and the recruitment of transcriptional regulators [5,23,91,92]. These modifications do not operate independently as they generate combinatorial chromatin states whose biological effects emerge from coordinated interactions rather than from the presence or absence of individual histone marks [91,92,93]. As a result, perturbation of a single chromatin-modifying enzyme may propagate throughout multiple regulatory pathways, ultimately producing genome-wide transcriptional responses that cannot be adequately explained by isolated changes in histone acetylation.

This complexity is further reflected in the highly context-dependent nature of curcumin-induced chromatin remodeling. The magnitude and direction of histone modifications vary according to cell lineage, differentiation state, metabolic profile and disease context. In greater detail, in cancer cells, where histone acetylation and chromatin organization are frequently disrupted [36,94], curcumin often promotes chromatin configurations associated with reduced proliferation [95,96,97], enhanced differentiation [17] and partial restoration of transcriptional homeostasis [98]. By contrast, in inflammatory or non-transformed cellular models, modulation of histone acetylation appears to predominantly affect cytokine expression [99], oxidative stress responses [100] and tissue-specific adaptive programs [87]. These observations suggest that curcumin does not impose a universal chromatin signature but instead remodels pre-existing regulatory networks according to the epigenetic state of each cellular system.

### 5.2. Curcumin and Histone Acetylation Beyond p300/CBP: Converging and Conflicting Evidence

The identification of p300/CBP as an epigenetic target represented a major milestone in understanding the chromatin-related activity of curcumin. However, subsequent investigations progressively demonstrated that histone acetyltransferases constitute only one component of a considerably broader network of chromatin-modifying enzymes influenced by this pleiotropic phytochemical. Rather than acting through a single molecular target, curcumin appears to modulate multiple epigenetic regulators controlling chromatin accessibility, histone turnover and transcriptional competence, thereby promoting coordinated remodeling of chromatin regulatory circuits rather than selective inhibition of individual enzymes [3,16,86,101].

Among these regulatory systems, histone deacetylases (HDACs) have received particular attention because of their central role in maintaining the dynamic equilibrium between histone acetylation and deacetylation. Liu and colleagues first demonstrated that curcumin suppresses the expression of HDAC1, HDAC3 and HDAC8 in Raji lymphoma cells while increasing histone H4 acetylation, leading to cell-cycle arrest and apoptosis [89]. Subsequently, Kang et al. reported that curcumin-induced histone hypoacetylation is closely linked to intracellular reactive oxygen species, highlighting the existence of functional crosstalk between oxidative stress and chromatin remodeling [90]. The two reports point in opposite directions (increased H4 acetylation with HDAC downregulation in Raji lymphoma cells versus ROS-linked hypoacetylation through reduced HAT activity in hepatoma cells). The difference may reflect cell type, concentration, exposure time or redox state, but these variables have not been compared systematically under matched experimental conditions. Sirtuins, the NAD^+^-dependent class III deacetylases, have also been implicated in the response to curcumin [102,103], although the evidence is limited and their contribution to the acetylation changes described above remains unclear [104]. Figure 3 additionally summarizes the evidence on curcumin and histone acetylation and highlights the unresolved questions. Overall, these findings indicate that curcumin can influence histone acetylation through more than one route, none of which has been shown to be primary (Figure 3).

Further mechanistic studies substantially strengthened this interpretation. As previously described, Marcu and co-workers demonstrated that curcumin not only inhibits the catalytic activity of p300/CBP but also promotes proteasome-dependent degradation of both p300 and CBP through a Michael acceptor-dependent mechanism, while sparing other acetyltransferases such as PCAF and GCN5 (Figure 3). These observations suggest that curcumin modifies the balance between histone acetylation and deacetylation at multiple regulatory levels, ultimately influencing chromatin accessibility and transcriptional competence.

Even so, histone acetylation alone cannot explain the diversity of transcriptional responses induced by curcumin. Histone modifications operate within a complex regulatory framework, in which acetylation, methylation, DNA methylation, chromatin accessibility and non-coding RNAs cooperate to establish cell-type-specific transcriptional programs [6,9,23,35,91,93]. As a result, perturbation of a single chromatin-modifying enzyme inevitably propagates through multiple epigenetic pathways, generating secondary molecular events that cannot be interpreted by examining individual histone marks alone.

Taken together, the enzyme-specific findings indicate that curcumin’s chromatin activity is not confined to p300/CBP and that HDACs, other acetyltransferases and redox-sensitive pathways have been implicated. Whether their combined perturbation, rather than any single target, determines the transcriptional outcome remains to be tested.

### 5.3. Genome-Wide Chromatin Profiling: Towards an Integrated View of Curcumin-Induced Histone Remodeling

Advances in epigenomic technologies have paralleled the progressive evolution of chromatin biology; a major turning point emerged with the development of chromatin immunoprecipitation coupled with high-throughput sequencing (ChIP-seq). This method finally enabled genome-wide mapping of histone modifications, transcription-factor occupancy, and chromatin-associated regulatory proteins [105]. This technology demonstrated that individual histone marks rarely function as isolated regulatory signals; instead, they contribute to combinatorial chromatin states associated with promoters, enhancers, super-enhancers, and other regulatory elements [34,93,105,106]. These observations fundamentally changed the interpretation of chromatin biology, shifting the focus from individual histone modifications toward coordinated epigenetic landscapes that integrate multiple regulatory inputs.

Subsequent technological innovations have further expanded our ability to investigate chromatin organization. The development of ATAC-seq enabled rapid genome-wide identification of accessible chromatin regions using minimal biological material, providing a direct measure of regulatory DNA accessibility [83]. More recently, CUT&RUN and CUT&Tag have substantially improved chromatin profiling by increasing signal-to-noise ratio, reducing cell number requirements and facilitating high-resolution mapping of histone modifications and chromatin-associated proteins, even in rare cellular populations [83]. Together, these approaches have transformed chromatin biology from locus-specific investigation into comprehensive reconstruction of regulatory landscapes at genome-wide resolution.

These technologies have revealed that chromatin accessibility, histone modifications and DNA methylation are highly interconnected rather than independent regulatory layers. Active promoters and enhancers are typically characterized by coordinated combinations of permissive histone marks, accessible chromatin and reduced promoter methylation, whereas transcriptionally repressed regions frequently display the opposite epigenetic configuration [9,33,34,35,46,91,92]. As a result, interpretation of individual histone modifications without considering the surrounding chromatin environment may provide an incomplete or even misleading view of epigenetic regulation.

Despite these technological advances, relatively few studies have applied genome-wide chromatin profiling to investigate the epigenetic effects of curcumin. Most published reports continue to focus on individual chromatin regulators or selected histone modifications, leaving unanswered how curcumin reshapes higher-order chromatin organization across the genome [3,4]. This limitation contrasts sharply with the increasing adoption of genome-wide methylome analyses discussed in previous sections and highlights one of the principal knowledge gaps in current curcumin epigenetics research.

Future investigations should, therefore, prioritize integrated experimental strategies combining ChIP-seq or CUT&Tag with ATAC-seq, DNA methylation profiling and transcriptomic analyses within the same biological models. Integration of these complementary datasets, particularly when combined with single-cell epigenomic technologies, would allow reconstruction of coordinated chromatin remodeling events rather than isolated epigenetic alterations [83,107]. Such multidimensional approaches are expected to determine whether specific histone modifications function as primary drivers or secondary consequences of curcumin’s broader pharmacology.

## 6. Non-Coding RNAs as Candidate Mediators of Curcumin-Associated Epigenetic Remodeling

The classical view of epigenetic regulation has changed with the discovery of non-coding RNAs (ncRNAs). These findings reveal that gene expression is controlled not only by DNA methylation and histone modifications but also by an extensive network of regulatory RNA molecules that coordinate transcriptional and post-transcriptional events [108,109,110]. Long treated as mere transcriptional noise with little biological meaning, ncRNAs are now recognized as regulators governing cellular identity, development, and disease pathways [111,112]. They have been implicated in the coordination of interactions among DNA methylation, chromatin remodeling, transcription-factor activity, and RNA stability [110,113]. This paradigm shift has considerably expanded the conceptual framework of epigenetics, moving beyond chromatin-associated modifications toward highly interconnected regulatory circuits in which RNA molecules participate in shaping the epigenetic landscape.

Among the different classes of ncRNAs, microRNAs (miRNAs), long non-coding RNAs (lncRNAs) and, more recently, circular RNAs (circRNAs) have emerged as the principal regulators of epigenetic homeostasis. Rather than acting independently, these RNA species cooperate through complex regulatory networks that influence virtually every level of gene regulation, including DNA methylation, histone modification, chromatin accessibility and messenger RNA turnover [110,113,114]. Notably, these interactions are bidirectional: ncRNAs regulate the expression and activity of epigenetic enzymes, while their own transcription is itself controlled by DNA methylation and chromatin organization. This arrangement generates intricate feedback mechanisms that contribute directly to transcriptional robustness and cellular plasticity [23,35].

This integrated view has important implications for understanding the biological activity of curcumin. While early studies predominantly focused on the direct modulation of DNA methyltransferases or histone-modifying enzymes, it has been proposed that part of the curcumin-induced transcriptional response may arise through ncRNA-mediated regulatory circuits, although most reports are correlative [55,115,116]. Because individual ncRNAs frequently regulate dozens or even hundreds of target genes, relatively modest changes in their expression can propagate across multiple signaling pathways, amplifying the biological consequences of primary epigenetic events. By contrast, epigenetic remodeling induced by curcumin may itself alter ncRNA expression, establishing reciprocal regulatory loops capable of reinforcing or stabilizing long-term transcriptional responses.

**Figure 4:**
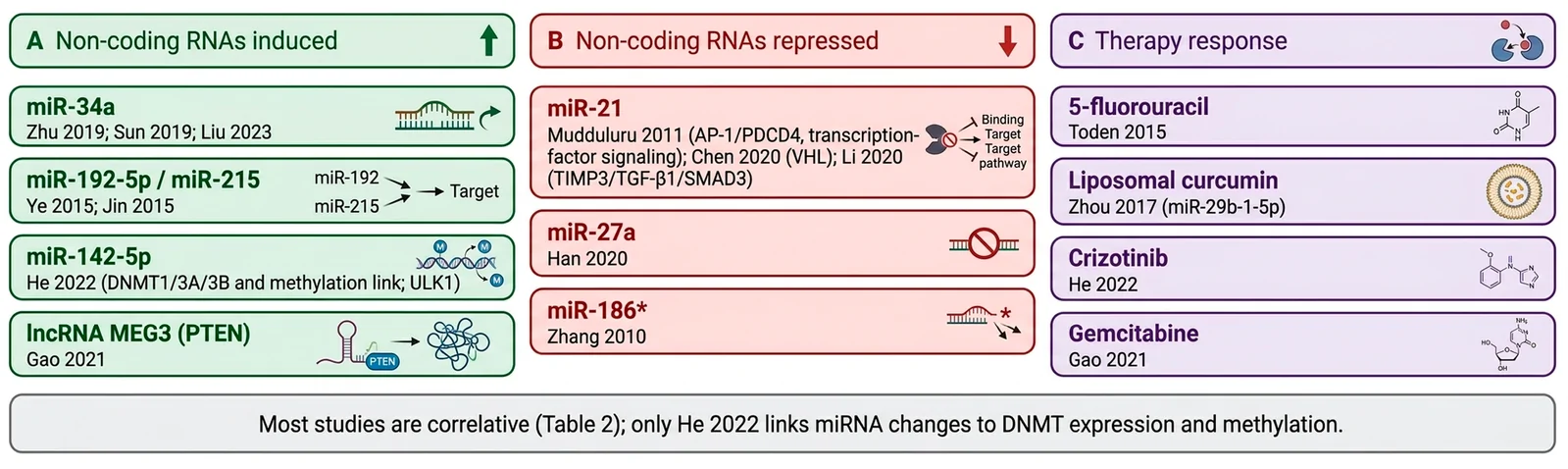
Non-coding RNAs reported to change after curcumin treatment: (**A**) induced, (**B**) repressed, (**C**) studies linking them to therapy response. Most studies are correlative, see Table 2 and Section 6 for details [31,54,117,118,119,120,121,122,123,124,125,126,127,128]. “*” It is the common nomenclature for antisense product associated with the MIR186.

Among ncRNAs, miRNAs have received by far the greatest attention in curcumin research. Table 2 summarizes the compound(s), experimental models, dose, exposure time and ncRNA reported in each study. Numerous studies have reported miRNAs involved in cell proliferation, apoptosis, epithelial–mesenchymal transition, inflammation, angiogenesis, cancer stemness and therapeutic resistance. In this line, one of the earliest mechanistic studies reported that curcumin reduced miR-21 transcription in colorectal cancer cells by interfering with AP-1 binding to the miR-21 promoter, resulting in restoration of the tumor suppressor PDCD4 and inhibition of tumor growth, invasion and metastasis [117] (Figure 4). Curcumin-related remodeling of miRNA expression was subsequently observed in multidrug-resistant A549/DDP lung adenocarcinoma cells, where modulation of miR-186 contributed to the pro-apoptotic response [118]. In NSCLC models, curcumin also induced miR-192-5p and miR-215 through a p53-dependent mechanism, leading to XIAP repression and apoptosis [119], while independent experiments linked miR-192-5p induction to suppression of PI3K/Akt signaling [120]. These results indicate that individual miRNAs can function as mechanistic intermediates rather than merely downstream biomarkers of curcumin exposure (Figure 4).

A similar regulatory logic emerges across different tumor contexts. Curcumin-mediated induction of miR-34a has been implicated in growth suppression in prostate and gastric cancer cells [121,122,123], whereas more recent genetic evidence demonstrated that activation of the ROS–KEAP1–NRF2 pathway induces miR-34a/b/c and contributes to apoptosis, senescence, mesenchymal-to-epithelial transition and suppression of colorectal cancer metastasis [124]. By contrast, curcumin can repress oncogenic miRNAs: downregulation of miR-27a contributed to inhibition of thymic carcinoma cell growth, migration and invasion [125], while repression of miR-21 has been functionally linked to the VHL axis in diffuse large B-cell lymphoma and to the TIMP3/TGF-β1/SMAD3 pathway in hepatocellular carcinoma [97,126] (Figure 4).

The ncRNA response to curcumin is also particularly relevant to therapeutic resistance. In chemoresistant colorectal cancer, curcumin restored sensitivity to 5-fluorouracil through miRNA-associated suppression of epithelial-to-mesenchymal transition [110], while liposomal curcumin remodeled miRNA expression in Adriamycin-resistant MCF-7 cells and altered their chemosensitivity, with miR-29b-1-5p emerging as one of the resistance-associated components [115]. These observations complement evidence that curcumin enhances crizotinib sensitivity through epigenetic restoration of miR-142-5p and modulation of the ULK1/autophagy axis [116] (Figure 4). Regulation is not restricted to miRNAs. In gemcitabine-resistant NSCLC, curcumin increased expression of the lncRNA MEG3 together with PTEN, and functional depletion of either component attenuated curcumin-induced growth inhibition and apoptosis [55]. Collectively, these studies support a model in which ncRNAs constitute functional nodes connecting curcumin-induced signaling and epigenetic remodeling to cell fate, metastatic plasticity and therapeutic responsiveness.

However, despite the large number of published reports describing individual miRNAs, the overall interpretation of these findings remains fragmented. Most investigations continue to examine single miRNAs or limited regulatory axes, whereas current evidence increasingly indicates that biological responses emerge from coordinated remodeling of entire ncRNA networks rather than isolated changes in individual transcripts. A further limitation is that miRNA mimic or inhibitor rescue experiments demonstrate functional involvement of a miRNA but not the origin of its altered expression; few studies tested whether the change arises from transcriptional (promoter methylation, histone marks, transcription-factor binding) or post-transcriptional mechanisms.

An additional challenge arises from the context dependency of ncRNA regulation. The same ncRNA may exert opposite biological functions depending on tissue type, developmental stage, metabolic state or disease context, while individual genes are frequently targeted by multiple ncRNAs acting simultaneously. As a result, assigning the biological effects of curcumin to modulation of a single miRNA often represents an oversimplification of considerably more complex regulatory processes. This complexity closely mirrors the conceptual evolution previously discussed for DNA methylation and histone modifications, further supporting the view that epigenetic regulation is better viewed as an integrated, multilayered network rather than as the sum of independent molecular mechanisms.

Collectively, these observations suggest that non-coding RNAs may contribute to curcumin-induced epigenetic remodeling. In most of the studies cited, however, miRNA changes were measured as changes in expression, and a causal link to DNA methylation or histone modification was shown only in a few cases (for example, miR-142-5p). Some examples, such as the AP-1-dependent repression of miR-21, reflect transcription-factor signaling rather than an epigenetic mechanism in the strict sense. Data on lncRNAs and circRNAs are currently very limited (MEG3 being the main example discussed here), and these classes are not analyzed further.

## 7. Future Directions in Curcumin Epigenetics: From Molecular Targets to Integrated Regulatory Networks

### 7.1. Beyond Single-Layer Epigenetics: Towards an Integrated Understanding of Curcumin Activity

Over the past two decades, substantial progress has been made in elucidating the epigenetic mechanisms underlying curcumin activity. Early studies focused on individual molecular targets—DNA methyltransferases, histone acetyltransferases and deacetylases, and selected non-coding RNAs—establishing curcumin as one of the most extensively investigated dietary phytochemicals capable of modulating gene expression through epigenetic regulation [4,28]. Despite this progress, current knowledge of curcumin-mediated epigenetic regulation remains intrinsically fragmented. DNA methylation, hydroxymethylation, histone modifications and non-coding RNAs continue to be studied largely as separate regulatory layers, using reductionist approaches centered on single targets or pathways [3,4,28]. This is at odds with growing evidence from systems epigenetics, indicating that biological phenotypes emerge from coordinated interactions among multiple regulatory layers—together with chromatin accessibility, enhancer activity, three-dimensional genome organization and transcription-factor networks—rather than from isolated alterations in a single mechanism [129,130].

This limitation is particularly relevant given curcumin’s marked context dependency: comparable treatments can elicit substantially different epigenetic and transcriptional responses depending on cell lineage, differentiation state, metabolic profile and disease context [17,131]. These differences may reflect distinct pre-existing chromatin configurations and regulatory network architectures that shape how individual cells interpret the same epigenetic perturbation [132,133]. The current data are more consistent with curcumin acting on several regulators and pathways than on a single epigenetic enzyme, although this has not been demonstrated at the network level. Future progress will accordingly depend less on identifying further molecular targets than on reconstructing the regulatory circuits that coordinate multiple epigenetic layers within the same biological system—an effort that will require experimental and computational frameworks able to integrate DNA methylation, chromatin organization, non-coding RNA activity, transcription and cellular phenotype [130,134]. Such an integrated view is expected to sharpen the mechanistic understanding of curcumin-induced epigenetic remodeling and to support the identification of biomarkers predictive of therapeutic response and disease susceptibility.

### 7.2. Integrating Multi-Omics Approaches to Decipher Curcumin Epigenetics

Translating this conceptual shift into practice requires moving from single-layer analyses to integrated multi-omics strategies capable of interrogating several regulatory layers within the same biological system. This is now technically feasible: genome-wide DNA methylation profiling can be combined with chromatin accessibility assays, histone modification mapping, transcriptomics and non-coding RNA profiling to reconstruct coordinated regulatory events rather than isolated molecular changes [134,135,136]. Such approaches are especially suited to curcumin, which rarely acts on a single target but instead modulates multiple pathways involved in inflammation, oxidative stress, metabolism and cell fate determination [3,4,17]. Multi-omics integration also helps distinguish primary epigenetic events from secondary transcriptional responses. Differentially methylated regions can be linked to concurrent changes in chromatin accessibility, histone marks and gene expression at the same genomic loci, while shifts in miRNA or lncRNA expression can be interpreted alongside promoter methylation and enhancer activation to reconstruct hierarchical regulatory circuits underlying specific phenotypes.

A further challenge is cellular heterogeneity: bulk analyses average signals across mixed populations, potentially masking cell-specific responses. Single-cell and multimodal omics technologies can instead resolve discrete subpopulations with distinct epigenetic trajectories following curcumin exposure [130,132,133], a particularly relevant issue in cancer, where curcumin differentially affects tumor cells, cancer stem cells, stromal and immune components, each with a distinct epigenetic landscape.

Ultimately, integrated multi-omics profiling should support the definition of composite molecular signatures—combining DNA methylation, chromatin state, non-coding RNA expression and transcriptional activity—rather than reliance on single genes or isolated modifications. Such multidimensional biomarkers are expected to offer greater predictive power for curcumin responsiveness, although no such signature has been developed for curcumin, and any candidate biomarker would require validation in independent cohorts and in clinical samples [130,133,134].

### 7.3. Computational Integration and Systems Biology in Curcumin Epigenetics

The scale and complexity of modern epigenomic datasets place the analytical burden beyond simple layer-by-layer interpretation: methylation, accessibility, transcriptomic and non-coding RNA data each carry limited biological meaning in isolation [6,130]. Realizing the integrative vision outlined above, therefore, requires computational frameworks—rooted in systems biology—capable of reconstructing the regulatory networks that link DNA methylation, hydroxymethylation, chromatin remodeling, transcription-factor activity and non-coding RNA regulation following curcumin exposure [137,138,139].

Rather than isolated differentially methylated genes or individual microRNAs, network-based analyses aim to identify coordinated molecular modules whose collective perturbation drives specific phenotypes. This approach may explain how curcumin consistently influences diverse processes—inflammation, oxidative stress, apoptosis, epithelial-to-mesenchymal transition, stemness—despite often modest effects on any single target. Machine learning offers a complementary route to interpreting these multidimensional datasets, enabling detection of hidden molecular patterns, classification of samples by integrated epigenetic signature, and prediction of functional interactions not evident from conventional statistics [134]. Applied to curcumin research, these methods could help identify epigenetic signatures associated with treatment responsiveness, disease subtype or therapeutic resistance, contributing to biomarkers with greater biological relevance than single molecular markers.

Critically, computational integration should be built into experimental design rather than applied post hoc: generating multiple omics layers from the same biological samples allows causal relationships to be tested, distinguishing epigenetic events directly induced by curcumin from secondary downstream responses, a distinction that will be essential for explaining curcumin’s context-dependent activity across tissues and pathological conditions.

Taken together, integrating experimental epigenomics with systems biology and computational modeling marks a shift from identifying isolated curcumin targets to reconstructing the dynamic regulatory networks governing cellular homeostasis, arguably the central conceptual challenge for the next generation of studies on this pleiotropic phytochemical.

## 8. Conclusions

The study of curcumin epigenetics has developed over the past two decades, in parallel with the broader development of epigenetic research itself. Initial investigations demonstrated that curcumin modulates individual components of the epigenetic machinery, including DNA methyltransferases, histone acetyltransferases, histone deacetylases and selected non-coding RNAs. More recent studies have progressively revealed that these mechanisms do not operate independently but instead constitute highly interconnected regulatory networks that collectively determine transcriptional plasticity, cellular identity and disease progression.

Despite these advances, important challenges remain. Much of the current literature continues to investigate isolated epigenetic mechanisms using reductionist experimental designs, limiting our ability to reconstruct the integrated molecular circuitry through which curcumin exerts its pleiotropic biological effects. Future progress will, therefore, depend on combining genome-wide experimental approaches with computational integration, enabling simultaneous interrogation of DNA methylation, hydroxymethylation, chromatin organization, non-coding RNAs and transcriptional dynamics within the same biological system.

In terms of clinical translation, this integrated framework may offer a useful conceptual basis for future studies of curcumin-based approaches, although its practical value remains to be established. Should multidimensional epigenetic signatures of disease progression, therapeutic responsiveness and patient stratification be validated, they could in principle support a more mechanism-informed use of nutraceuticals than empirical supplementation. This remains a prospective consideration, contingent on biomarker validation and clinical evidence not yet available for curcumin. Clinical translation of the epigenetic effects of curcumin is currently limited by its low systemic bioavailability and by the uncertainty over whether the concentrations used in vitro can be reached in vivo (Section Pharmacological Constraints on the Interpretation of Epigenetic Effects). Epigenetic signatures may eventually help to stratify responders in trials of curcumin or its formulations, but at present, there is no clinical evidence supporting this use.

In conclusion, the available evidence does not support describing curcumin as a specific epigenetic drug. The most robust findings are cell-line-specific, bidirectional changes in DNA methylation and effects on chromatin-modifying enzymes and non-coding RNAs, mostly obtained at micromolar concentrations. Whether these changes are direct, indirect, or consequences of redox stress remains open, and integrated multi-omics studies with native curcumin at attainable concentrations are required to address it.

## Figures and Tables

**Table 1:** Experimental studies cited in the review investigating epigenetic effects of curcumin, curcuminoids and chemical derivatives in different experimental settings and models.

	Year	Experimental Model	Treatment	Dose	Method	Main Finding
Liu et al.	2009	MV4-11 leukemia cells; bacterial CpG methyltransferase M.SssI (cell-free); DNMT1 docking	Commercial curcumin (mixture of curcumin, demethoxycurcumin and bisdemethoxycurcumin) in cells; commercial and HPLC-purified compounds in the M.SssI assay	M.SssI IC50 = 30 nM; hexahydrocurcumin inactive up to 100 µM; MV4-11 cells: 0, 1, 3 and 30 µM for 72 h	Methyltransferase activity; global DNA methylation (LC–MS/MS); molecular docking	Curcumin inhibited M.SssI activity and reduced global DNA methylation by about 15–20% at 3 and 30 µM (unchanged at 1 µM); direct inhibition of human DNMT1 was inferred mainly from docking.
Shu et al.	2011	LNCaP human prostate cancer cells	Native curcumin	5 µM; 5 days (initial analyses) and 7 days (main experiments); medium changed every 2 days	NEUROG1 promoter CpG methylation; DNMT1; local chromatin marks	Reduced NEUROG1 promoter methylation and restored expression, with accompanying changes in DNMT1 and local chromatin regulation.
Du et al.	2012	MCF-7 and MDA-MB-231 breast cancer cells; MCF-7 xenograft (nude mice)	Native curcumin	10 and 20 µM; 72 h; xenograft: 100 mg/kg i.p., 5 d/week, 4 weeks	RASSF1A promoter methylation; methyltransferase activity; DNMT1	RASSF1A reactivation was associated with reduced methylation and DNMT1 expression/activity, involving NF-κB/Sp1 regulation.
Yu et al.	2013	AML cell lines; primary AML cells ex vivo; murine leukemia model	Native curcumin	10–20 µM (cell lines), 10 µM (primary AML cells); 24–72 h; xenograft: 100 mg/kg i.p., 5 d/week, 4 weeks	DNMT1 (immunoblot, RT-qPCR); p65/Sp1 binding; p15INK4B promoter methylation (LC-MS/MS)	Curcumin downregulated DNMT1 via p65/Sp1, reduced p15INK4B promoter methylation (about 40%), reactivated p15INK4B and inhibited MV4-11 tumor growth in mice.
Link et al.	2013	HCT116, RKO and HT29 human colorectal cancer cells	Native curcumin	7.5 μM (HCT116); 10 μM (RKO, HT29); 6 d and 240 d	Illumina HumanMethylation27 array; LINE-1 pyrosequencing; gene-expression arrays	Curcumin produced selective hyper- and hypomethylation, especially at partially methylated loci, rather than non-selective global hypomethylation.
Hassan et al.	2015	Human leukemia cell lines (including CEM, Kasumi-1, BV-173)	Native curcumin and dimethoxycurcumin	1–2 µM for 72–168 h; 5–10 µM for 72–120 h (CEM)	DNMT isoforms; p15 and CDH1 promoter methylation by pyrosequencing; gene re-expression	Both compounds altered epigenetic regulators and reactivated genes, but gene re-expression occurred with little or no corresponding promoter demethylation.
Jiang et al.	2015	A549 and H460 lung cancer cells; xenograft (nude mice)	Native curcumin	1 and 5 µM, 24 h; xenograft: 40 mg/kg i.p. every other day for 4 weeks	RARβ promoter DNA methylation and expression	Curcumin reactivated RARβ in association with reduced promoter methylation.
Liu et al.	2017	MDA-MB-361 human breast cancer cells; xenograft (nude mice)	Native curcumin	10, 20 and 40 µM, 48 h; xenograft: 50 mg/kg intratumoral, once a week for 4 weeks	DLC1 promoter methylation and expression	Curcumin reduced DLC1 promoter methylation, restored DLC1 expression and inhibited breast-cancer cell growth.
Chen et al.	2020	SW480 human colorectal cancer cells	Native curcumin	0.1–0.4 µM	DNMT1/DNMT3a; CDX2 promoter methylation; CDX2/Wnt/β-catenin; EMT	Curcumin reduced CDX2 promoter methylation and was associated with suppression of EMT through the CDX2/Wnt/β-catenin axis.
Tong et al.	2020	MGC-803 human gastric cancer cells	Native curcumin	10–60 µM, 24–72 h (viability); 20 µM for 12–24 h (mechanistic assays)	DNMT1; DNA methylation; DNA-damage response; re-expression of silenced genes	Curcumin-induced demethylation was linked to activation of the DNA-damage response and reduced DNMT1 expression.
He et al.	2022	A549 and H460 non-small-cell lung cancer cells	Curcumin + crizotinib	Curcumin (90% purity) alone IC50 at 48 h: 26.8 µM (A549), 27.7 µM (H460); crizotinib IC50 15.3 and 16.7 µM; concentrations used for miR-142-5p, DNMT and methylation assays: 15–30 µM	miR-142-5p epigenetic regulation; DNMT1/DNMT3A/DNMT3B; ULK1/autophagy	Curcumin increased crizotinib sensitivity through epigenetic restoration of miR-142-5p and suppression of the miR-142-5p/ULK1 autophagy axis.
Nirgude et al.	2022	MDA-MB-231 human triple-negative breast cancer cells	Synthetic curcumin derivatives ST08 and ST09	ST08 75 nM; ST09 50 nM; 48 h (IC50 40–80 nM)	Whole-genome bisulfite sequencing (WGBS) + RNA-seq	ST08 and ST09 caused distinct combinations of hyper- and hypomethylation and compound-specific transcriptional signatures; ST08 favored ECM-related pathways and ST09 cell-cycle pathways.
Poma et al.	2024	Doxorubicin-resistant MCF-7 human breast cancer cells	Native curcumin	30 µM; 24 h	RRBS (2 biological replicates per condition); RT-qPCR of selected genes; P-gp Western blot; NF-κB DNA-binding assay	Predominantly demethylating effect at specific loci (83% of DMRs hypomethylated; rDNA, Wnt, chromatin-regulator and ncRNA loci); 64% of the demethylated DMRs were hypermethylated in resistant versus parental cells.
Balasubramanyam et al.	2004	Cell-free p300/CBP and chromatin-transcription assays; HeLa and 293T cells; SupT1/H9 HIV co-culture	Native curcumin	Cell-free p300 HAT assay: IC50 about 25 µM; HeLa cells: 75–100 µM, 24 h; 293T cells: 100 µM, 24 h	p300/CBP histone acetyltransferase activity; histone/non-histone acetylation; chromatin transcription	Curcumin inhibited p300/CBP acetyltransferase activity in vitro (cell-free assays) and repressed HAT-dependent acetylation and chromatin transcription.
Marcu et al.	2006	Purified p300; SKBr3 breast cancer cells; PC3-M prostate cancer cells; peripheral blood lymphocytes	Native curcumin	Cell-free p300 assay: 10–100 µM (maximal inhibition 40–100 µM; 80 µM pre-incubation); SKBr3 cells: up to 30 µM, 8 h; PC3-M cells and lymphocytes: 6 h with MS-275, curcumin concentration 30 µM	p300/CBP HAT activity and protein abundance	Curcumin inhibited p300 HAT activity and promoted proteasome-dependent depletion of p300/CBP in a Michael acceptor-dependent manner.
Liu et al.	2005	Raji human B-cell non-Hodgkin lymphoma cells	Native curcumin	36-h IC50 = 24.1 µM; HDAC and acetyl-H4 analyses: 6.25, 12.5 and 25 µM for 24 h	HDAC1/HDAC3/HDAC8 expression; histone H4 acetylation; cell-cycle/apoptosis assays	Curcumin suppressed class I HDAC expression, altered histone acetylation and inhibited Raji-cell proliferation.
Kang et al.	2005	Hep3B human hepatoma cells	Native curcumin	Hep3B cells: no change in histone acetylation at 20 µM or less, decrease at 25 µM or more (20 h); time course at 50 and 100 µM	Histone acetylation; reactive oxygen species	Curcumin-induced histone hypoacetylation was linked to intracellular ROS, supporting crosstalk between oxidative stress and chromatin regulation.

**Table 2:** Studies reporting changes in non-coding RNA expression after curcumin treatment and their reported functional consequences. Most studies are correlative and do not demonstrate an epigenetic mechanism.

	Year	Model	Treatment	Dose	ncRNA	Methods	Main Finding
Mudduluru et al.	2011	RKO and HCT116 colorectal cancer cells; CAM in vivo metastasis model	Native curcumin	Dose-dependent; 24 h for several cellular assays	miR-21/AP-1/PDCD4	qRT-PCR; promoter analysis; AP-1 binding; functional invasion/metastasis assays	Curcumin reduced miR-21 transcription through inhibition of AP-1 promoter occupancy, restored PDCD4 and suppressed growth, invasion and metastasis.
Zhang et al.	2010	A549/DDP multidrug-resistant human lung adenocarcinoma cells	Native curcumin	5–40 μM tested	miR-186*; apoptosis/multidrug resistance	miRNA-expression analysis; miRNA inhibition/overexpression; apoptosis assays	Curcumin altered miRNA expression and downregulated miR-186*; functional manipulation linked this change to curcumin-induced apoptosis in resistant cells.
Ye et al.	2015	H460, A427, A549 and H1299 NSCLC cells; in vivo tumor model	Native curcumin	5–30 μM tested; 15 μM used for subsequent mechanistic experiments	p53–miR-192-5p/miR-215–XIAP	miRNA microarray; qPCR; genetic p53 manipulation; apoptosis assays; in vivo analysis	Curcumin induced miR-192-5p/215 in a p53-dependent manner; these miRNAs mediated apoptosis through XIAP suppression.
Toden et al.	2015	5-FU-resistant colorectal cancer models	Native curcumin + 5-fluorouracil	5–30 μM tested; 24 h; 15 μM used for synergical experiments	miRNA-mediated EMT/chemoresistance	miRNA analysis; EMT markers; chemosensitivity and functional assays	Curcumin restored 5-FU sensitivity in chemoresistant colorectal cancer through miRNA-associated suppression of EMT.
Jin et al.	2015	Human NSCLC cells (including A549)	Native curcumin	10–40 μM; 24 h	miR-192-5p/PI3K-Akt	qPCR; proliferation/apoptosis assays; pathway analysis	Curcumin upregulated miR-192-5p, suppressed PI3K/Akt signaling and inhibited proliferation while promoting apoptosis.
Zhou et al.	2017	Adriamycin-resistant MCF-7/Adr breast cancer cells	Liposomal curcumin	6,8 µM, 13,58 µM, 27 µM in combination with Adriamycin; 48 h	miRNA/mRNA expression; miR-29b-1-5p; chemoresistance	miRNA and mRNA microarrays; qRT-PCR; chemosensitivity assays	Liposomal curcumin remodeled miRNA expression and altered Adriamycin sensitivity; miR-29b-1-5p emerged as a resistance-associated mediator.
Zhu et al.	2019	Three prostate cancer cell lines	Native curcumin	1–10 μM; 4 days	miR-34a/β-catenin/c-Myc	qPCR; miR-34a inhibition; proliferation and cell-cycle analyses	Curcumin increased miR-34a and suppressed the β-catenin/c-Myc axis; miR-34a inhibition attenuated the antiproliferative response.
Sun et al.	2019	SGC-7901 gastric cancer cells; tumor tissue analyses	Native curcumin	50 μM; 1–4 days	miR-34a/BCL-2/CDK4/cyclin D1	miR-34a agomir/antagomir; proliferation, migration, invasion, apoptosis and cell-cycle assays	Curcumin increased miR-34a; antagonizing miR-34a weakened curcumin-induced growth inhibition and apoptosis.
Chen et al.	2020	SU-DHL-8 diffuse large B-cell lymphoma cells	Native curcumin	0–60 μM; 24 h for migration/invasion; 20 μM used in mechanistic rescue experiments	miR-21/VHL	qRT-PCR; luciferase; RIP; MTT; flow cytometry; migration/invasion assays	Curcumin dose-dependently suppressed miR-21; functional rescue experiments supported a miR-21/VHL mechanism for antiproliferative and pro-apoptotic effects.
Li et al.	2020	HepG2 and HCCLM3 hepatocellular carcinoma cells; HepG2 xenografts in nude mice	Native curcumin	Dose-dependent; 24 h in cellular expression experiments	miR-21/TIMP3/TGF-β1-SMAD3	RT-qPCR; western blot; dual-luciferase; proliferation/apoptosis; xenograft analysis	Curcumin downregulated miR-21, increased TIMP3 and inhibited TGF-β1/SMAD3 signaling in vitro and in vivo.
Han et al.	2020	TC1889 thymic carcinoma cells	Native curcumin	24 h	miR-27a/mTOR/Notch1	qPCR; miR-27a mimic; viability, apoptosis, migration/invasion; western blot	Curcumin downregulated miR-27a; miR-27a overexpression reversed curcumin effects, linking the response to mTOR and Notch1 suppression.
Gao et al.	2021	Gemcitabine-resistant NSCLC cells; xenograft model	Native curcumin	50, 100 μM; 48 h,	lncRNA MEG3/PTEN	qRT-PCR; knockdown/overexpression; proliferation/apoptosis; western blot; IHC; xenografts	Curcumin upregulated MEG3 and PTEN; knockdown of either component attenuated curcumin-mediated growth suppression and apoptosis.
He et al.	2022	A549 and H460 NSCLC cells	Curcumin + crizotinib	10, 30 μM; 24 h	miR-142-5p/DNMT1-DNMT3A-DNMT3B/ULK1-autophagy	DNA methylation/miRNA expression; DNMT analyses; functional chemosensitivity/autophagy assays	Curcumin enhanced crizotinib sensitivity through epigenetic restoration of miR-142-5p and modulation of the miR-142-5p/ULK1 autophagy axis.
Liu et al.	2023	Isogenic colorectal cancer cells deficient for p53 and/or miR-34; NOD/SCID lung-metastasis model	Native curcumin	5–40 μM; 24–72 h	ROS–KEAP1–NRF2–miR-34a/b/c	qPCR; qChIP; genetic deletion; migration/invasion/apoptosis; longitudinal in vivo imaging	Curcumin activated NRF2-dependent miR-34a/b/c expression, driving apoptosis/senescence, MET and suppression of lung metastasis.

## Data Availability

No new data were created or analyzed in this study. Data sharing is not applicable to this article.
